# Systematic Comparison of Commercial Hydrogels Revealed That a Synergy of Laminin and Strain-Stiffening Promotes Directed Migration of Neural Cells

**DOI:** 10.1021/acsami.2c20040

**Published:** 2023-03-06

**Authors:** Flavia Millesi, Sascha Mero, Lorenz Semmler, Anda Rad, Sarah Stadlmayr, Anton Borger, Paul Supper, Maximilian Haertinger, Leon Ploszczanski, Ursula Windberger, Tamara Weiss, Aida Naghilou, Christine Radtke

**Affiliations:** † Research Laboratory of the Department of Plastic, Reconstructive and Aesthetic Surgery, Medical University of Vienna, Vienna 1090, Austria; ‡ Austrian Cluster for Tissue Regeneration, Vienna 1200, Austria; § Institute for Physics and Materials Science, University of Natural Resources and Life Sciences, Vienna 1190, Austria; ∥ Decentralized Biomedical Facilities, Core Unit Laboratory Animal Breeding and Husbandry, Medical University Vienna, Vienna 1090, Austria; ⊥ Department of Physical Chemistry, University of Vienna, Vienna 1090, Austria; # Department of Plastic, Reconstructive and Aesthetic Surgery, Medical University of Vienna, Vienna 1090, Austria

**Keywords:** peripheral nerve regeneration, tissue engineering, biomaterials, directionality, mechanobiology, stiffness, Schwann cells

## Abstract

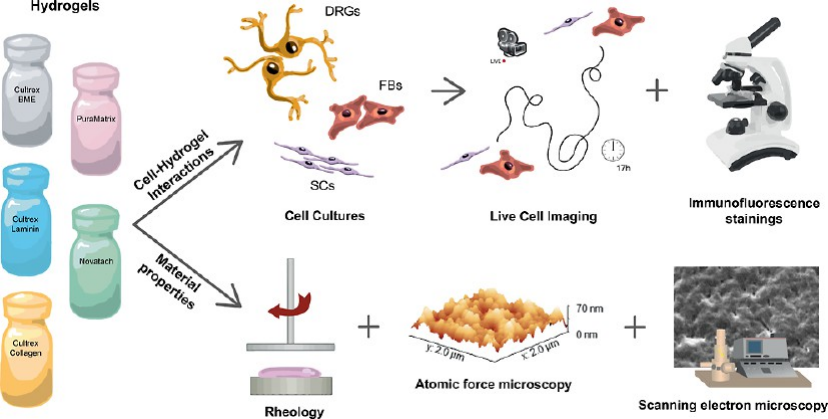

Hydrogels have shown potential in replacing damaged nerve tissue, but the ideal hydrogel is yet to be found. In this study, various commercially available hydrogels were compared. Schwann cells, fibroblasts, and dorsal root ganglia neurons were seeded on the hydrogels, and their morphology, viability, proliferation, and migration were examined. Additionally, detailed analyses of the gels’ rheological properties and topography were conducted. Our results demonstrate vast differences on cell elongation and directed migration on the hydrogels. Laminin was identified as the driver behind cell elongation and in combination with a porous, fibrous, and strain-stiffening matrix structure responsible for oriented cell motility. This study improves our understanding of cell–matrix interactions and thereby facilitates tailored fabrication of hydrogels in the future.

## Introduction

Tissue engineering and regenerative medicine (TERM) designed hydrogels to mimic the extracellular matrix (ECM) and to thereby replace injured tissue.^1^ While harvest and decellularization of the native ECM provides a more ideal scaffolding for this purpose, batch-to-batch variations and low production rates limit their applicability.^2^ Furthermore, the decellularization processes can be deficient, resulting in strong inflammatory responses.^3^ Hence, hydrogels present an attractive alternative to autologous material in the form of an artificial ECM with more control over the mechanical, topological and biochemical properties.^4^

Hydrogels are water-swollen, three-dimensional networks of hydrophilic polymers.^5^ Upon physical or chemical stimuli such as temperature changes, these polymers crosslink, thereby enabling the hydrogel to maintain its structure while containing large amounts of water.^6^ They have a wide variety of applications ranging from drug delivery to tissue replacement, and their ideal characteristics are highly dependent on the intended use.^5^ Even within TERM, various hydrogels are required depending on their site of deployment, such as bone, cartilage, and muscle tissue.^7^ Another important application of hydrogels is supporting the regeneration of the peripheral nervous system (PNS).^8^ The current gold standard to reconstruct lost nervous tissue is the transplantation of an intact donor nerve in order to recover essential sensory and/or motor functions.^9^ Nevertheless, this procedure necessitates the harvest of autologous material which, besides longer operating time and a second incision, entails donor site morbidity.^10^ Over the past decades, nerve tissue engineering has made major advances in the development of alternatives to the nerve autograft.^11^ Artificial nerve guidance conduits (NGCs), tubes inserted at the injury site,^12^ circumvent donor site morbidity as well as the autografts’ limited availability.^13^ NGCs have already been employed clinically and resulted in satisfactory outcomes in nerve lesions up to 3.0 cm.^14^ However, hollow NGCs remain inferior to nerve autografts when used for long-distance nerve gaps.^15^ Filling materials, such as hydrogels, improve NGCs by preventing the tubular device from collapsing and simultaneously offering a three-dimensional matrix that facilitates cellular integration into the conduit.^16^

Peripheral nerve regeneration (PNR) requires specific cellular components at the injury site.^17^ To bridge the gap between the proximal and distal nerve stump, Schwann cells (SCs), the principal glial cells in the PNS, were shown to change their phenotype into a repair status and clear the cellular debris together with macrophages. At the cleared site of injury, SCs increase the production of ECM and cell adhesion molecules and align to form bands of Büngner, which guide the regrowing axons toward the target organ.^18^ Fibroblasts (FBs) provide further assistance in this regenerative process by synthesizing and remodeling the ECM surrounding the regrowing nerve fibers.^19^ In addition to the regenerating neurons, SCs and FBs are therefore the crucial components of a successful reinnervation after peripheral nerve injury (PNI).^20^ Consequently, understanding the response of these three cell types to hydrogels is a prerequisite for the successful implementation of this NGC filling material in nerve reconstruction.

To this end, this study first systematically compared three commercially available hydrogels advertised for peripheral nerve repair, namely, PuraMatrix (Corning), Cultrex basement membrane extract (Cultrex BME) (Trevigen), and Novatach (NovaMatrix). These hydrogels were chosen due to their prominently different source material, allowing for a systematic comparison. PuraMatrix is a synthetic polypeptide hydrogel consisting of 99% water and 16 amino acids.^21^ Cultrex BME is a basement membrane hydrogel purified from a mouse Engelbreth–Holm–Swarm tumor similar to the more well-known Matrigel (Corning; Corning, NY, USA).^22^ It consists of laminin I, type IV collagen, entactin, and heparan sulfate proteoglycan.^23^ Novatach is a hydrogel formed out of brown algae-derived alginate coupled with the fibronectin-derived adhesion peptide arginine glycine aspartic acid (RDG).^24^

SCs, FBs, and dorsal root ganglia (DRG) cultures were seeded on the hydrogels followed by detailed *in vitro* analyses including live cell imaging and cell tracking as well as multicolor immunofluorescence stainings and confocal microscopy. The experiments indicated that the hydrogel Cultrex BME induced two important features for PNR, a more elongated SC morphology, and a directed cell migration. For elucidating the reasons behind this favorable effect of Cultrex BME, follow-up experiments using the two additional hydrogels that make up Cultrex BME [Cultrex Laminin I (Cultrex Laminin) (Trevigen) and Cultrex Rat Tail Collagen I (Cultrex Collagen) (Trevigen)] as well as a laminin concentration series were performed. In addition, scanning electron (SEM) and atomic force microscopy (AFM) as well as rheological analyses were conducted to investigate the material properties of the hydrogels. Laminin was identified as the main reason behind increased elongation of SCs. Our experiments further revealed a possible synergy between laminin and a porous and fibrous morphology of hydrogels as well as strain-stiffening for directed cell migration. These findings are invaluable for the future of tailored materials and pave the way for a target-oriented manufacturing of tissue replacements.

## Results

### Cultrex BME Endorses Schwann Cell to Neurite Alignment

**Figure 1:**
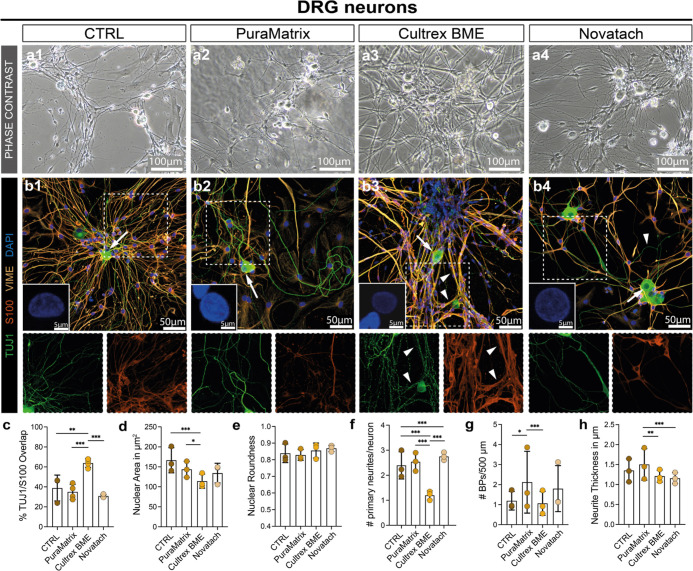
DRG neuron cultures seeded in hydrogels and CTRL. (a) Phase contrast micrographs of DRG neuron cultures on (1) PDL/laminin-coated cell culture dishes (CTRL) and in hydrogels (2) PuraMatrix, (3) Cultrex BME, and (4) Novatach. (b) Representative confocal micrographs of cultures stained for TUJ1 in green, S100 in red, vimentin (VIME) in orange, and DAPI in blue. Images in the left corner are representative micrographs of DAPI^+^ cell nuclei of a single DRG neuron, indicated by white arrows. Enlargements of the neurite networks show TUJ1 and S100 stainings to assess neurite/SC co-localization indicated by white arrowheads. (c) Diagram depicts the mean ± SD percentage of S100 (SC) and TUJ1 (DRG neurons) overlap (*n* = 3). (d) Diagram depicts the mean ± SD nuclei size of DRG neurons in μm^2^ for the four conditions (*n* = 3). (e) Diagram depicts the mean ± SD nuclei roundness (*n* = 3). (f) Diagram depicts the mean ± SD number of primary neurites (*n* = 3). (g) Diagram depicts the mean ± SD number of BPs per 500 μm neurite length (*n* = 3). (h) Diagram depicts the mean ± SD neurite thickness in μm (*n* = 3). * *p*-value < 0.05, ** *p*-value < 0.01, *** *p*-value < 0.001.

Successful nerve recovery is defined by the re-grown nerve fibers that re-innervate the target organ.^25^ It is therefore essential to evaluate the effect of regeneration-supporting matrices on neurite outgrowth. For this, we analyzed DRG cultures seeded on the three hydrogels; PuraMatrix, Cultrex BME, and Novatach. Poly-d-lysine (PDL)/laminin-coated cell culture dishes served as control (CTRL) since this is one of the standard coatings for DRG neurons *in vitro*.^26^ Similar to the CTRL, all three hydrogels promoted DRG neuron adhesion and successful neurite outgrowth (Figure 1a). Notably, in Cultrex BME (Figure 1a3), the neuron extensions appeared thicker compared to the other hydrogels and the CTRL.

Immunofluorescence stainings of DRG cultures revealed that, besides TUJ1^+^ DRG neurons, a high proportion of cells in cultures were S100^+^ SCs and, especially in PuraMatrix, S100^–^ FBs (Figure 1b2). Interestingly, the thicker appearing neurites in Cultrex BME, visible in the phase contrast micrographs (Figure 1a3), represented SCs aligning to the neuron processes (arrowheads, Figure 1b3). This was confirmed by comparing the percentage of TUJ1/S100 co-localization in the immunofluorescence micrographs, which was significantly higher in Cultrex BME compared to the other groups (Figure 1c).

Because cell morphology is an indicator for cellular behavior, we next quantified morphological parameters such as nuclear roundness, number of primary neurites, and neurite branching points.^27^ A similar cellular appearance of DRG neurons was seen in CTRL and PuraMatrix (Figure 1d–g). In Cultrex BME, the neurons had significantly smaller nuclei than the neurons in the CTRL and in PuraMatrix (Figure 1d). However, there was no significant difference in nuclei roundness (Figure 1e). Although not evident from the phase contrast images due to the high overlap of SCs and neurites, the immunofluorescence stainings show that the neurons in Cultrex BME had significantly fewer primary neurites than the neurons in the CTRL, PuraMatrix or Novatach (Figure 1f). The neurons in Cultrex BME mostly had only one primary neurite extending from their cell body, whereas the DRG neurons in the CTRL, PuraMatrix, and Novatach had a mean primary neurite number between two and three (Figure 1f). Furthermore, there were significant differences in the mean number of BPs per 500 μm of neurite length (Figure 1g). The neurons in Cultrex BME had significantly less BPs per 500 μm compared to the number of BPs in PuraMatrix. In Novatach, the neurons had significantly more primary neurites than the neurons in the CTRL (Figure 1f) and less BPs per 500 μm than the neurons in PuraMatrix (Figure 1g). There were no significant differences between the neurite thickness in CTRL, Cultrex BME and Novatach (Figure 1h). However, the neurons in PuraMatrix had significantly thicker neurites in comparison to the neurites in the CTRL and Cultrex BME (Figure 1h). Thus, Cultrex BME induced the alignment of SCs to neurites and promoted the growth of only one primary neurite, while neurites were thicker in PuraMatrix. All data can be found in Table S2.

### Cultrex BME Promotes Schwann Cell and FB Elongation

The SCs’ remarkable ability to transdifferentiate into a repair phenotype after PNI goes hand in hand with distinct changes in their morphology such as increased cell elongation.^18^ Hydrogels used in PNR must therefore support this phenotype. Hence, detailed morphological assessments of SCs and FBs were performed by phase contrast as well as confocal microscopy.

**Figure 2:**
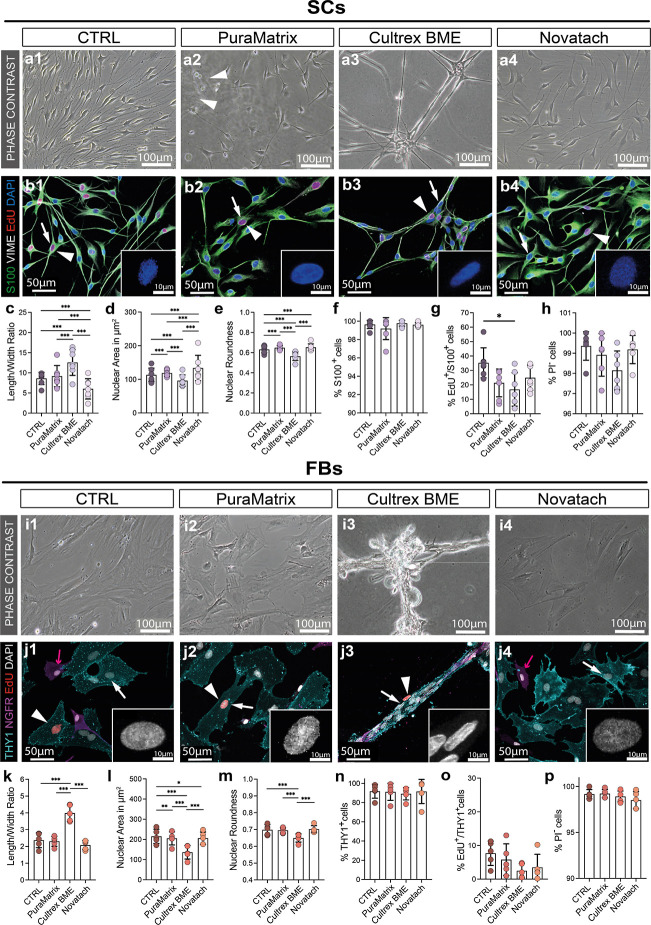
Evaluation of SC and FB morphology, proliferation, and viability. (a) Representative phase contrast micrographs of SC cultures on (a1) poly-l-lysine (PLL)/laminin-coated cell culture dishes (CTRL), (a2) PuraMatrix, (a3) Cultrex BME, and (a4) Novatach. Hydrogel residues of PuraMatrix were free-floating in the medium [arrowheads in (a2)]. (b) Representative confocal micrographs of SCs stained for S100 in green, VIME in gray, EdU in red, and DAPI in blue. The arrowheads indicate proliferating SCs. Small images in the right corner are representative micrographs of a DAPI^+^ cell nucleus of a single SC, indicated by white arrows. (c) Diagram depicts the mean ± SD length/width ratio of SCs on PLL/laminin (CTRL) and hydrogels (*n* = 7). (d) Diagram depicts the mean ± SD nuclei size of SCs in μm^2^ for the four groups (*n* = 7). (e) Diagram depicts the roundness of SC nuclei on PLL/laminin and hydrogels ± SD (*n* = 7). (f) Diagram shows the mean ± SD percentage of S100^+^ cells (SCs) for each group (*n* = 6). (g) Diagram depicts the mean ± SD percentage of S100^+^/EdU^+^ cells (proliferating SCs) in each condition (*n* = 7). (h) Diagram depicts the percentage of SCs negative for propidium iodide (PI) in each condition ± SD (*n* = 6). (i) Representative phase contrast micrographs of FB cultures on (i1) uncoated cell culture wells (CTRL), (i2) PuraMatrix, (i3) Cultrex BME, and (i4) Novatach. (j) Representative confocal micrographs of FBs stained for THY1 in cyan, NGFR in pink, EdU in red, and DAPI in gray. The arrowhead indicates proliferating FBs, and the pink arrows indicate NGFR^+^ SCs. Small images in the right corner of the image are representative micrographs of a DAPI^+^ cell nucleus of a single FB, pointed out by the white arrows. (k) Diagram depicts the mean ± SD length/width ratio of FBs in CTRL and hydrogels (*n* = 5). (l) Diagram depicts the mean ± SD nuclei size of FBs in μm^2^ (*n* = 5). (m) Diagram depicts the mean ± SD roundness of FB nuclei in CTRL and hydrogels (*n* = 5). (n) Diagram shows the percentage of THY1^+^ cells (FBs) for each group (*n* = 5). (o) Diagram depicts the mean ± SD number of THY1^+^/EdU^+^ cells (proliferating FBs) (*n* = 5). (p) Diagram depicts the mean ± SD percentage of FBs negative for PI (alive FBs) (*n* = 5). * *p*-value < 0.05, ** *p*-value < 0.01, *** *p*-value < 0.001.

Upon seeding into hydrogels, striking morphological differences were observed (Figure 2a). In PuraMatrix, the SCs displayed their spindle-shaped morphology, but some SCs appeared more spherical and rounder in shape with only short extensions (Figure 2a2). However, immunofluorescence analyses showed that there were no significant differences between the length/width ratios of SCs in CTRL compared to the SCs in PuraMatrix (Figure 2c). In contrast, the nuclei of SCs in PuraMatrix were significantly larger and rounder than the CTRL (Figure 2b2,d,e). Interestingly, the PuraMatrix gel showed occasional detachment from the well as pointed out by the arrowheads in Figure 2a2. The SCs in Cultrex BME formed interconnected star-like structures over multiple *z*-planes thereby using the gel’s three-dimensional matrix (Figure 2a3). The basement membrane hydrogels also induced significantly increased elongation as seen by the SCs in Cultrex BME having the highest length/width ratio (Figure 2c). Moreover, the SC nuclei in Cultrex BME were the smallest and least round, significantly differing from all other conditions (Figure 2b3,d,e). In Novatach, SCs showed a rather broad and flat morphology with the lowest length/width ratio (Figure 2c), most deviating from the typical spindle shape (Figure 2a4). Moreover, the SC nuclei in Novatach were significantly larger and rounder than the SC nuclei in CTRL (Figure 2b4,d,e). Immunofluorescence stainings as seen in Figure 2b visualized that SCs in Cultrex BME proliferated significantly less than the SCs in CTRL. Arrowheads in Figure 2b mark S100^+^/EdU^+^ proliferating SCs. No significant differences were observed in culture composition, proliferation rate, and viability between SCs in CTRL, SCs in PuraMatrix, and SCs in Novatach (Figure 2f–h).

FBs in PuraMatrix (Figure 2i2) had a similar planar and flat morphology as the FBs on uncoated wells (CTRL, Figure 2il), and there were no significant differences in the length/width ratio compared to the FBs in CTRL (Figure 2k). Moreover, FB nuclei in PuraMatrix were significantly smaller, but there were no significant differences in nuclei roundness between the cells in CTRL and the cells in PuraMatrix (Figure 2j2,l,m). Similar to the SC cultures in Cultrex BME, FBs formed star-like structures in the basement membrane hydrogel, spanning over multiple *z*-planes (Figure 2i3,j3) and their nuclei were significantly smaller and less round compared to the other conditions (Figure 2j3,l,m). In Novatach, FBs exhibited a similar morphology to the FBs in CTRL (Figure 2i4) and there were no significant differences regarding cell length/width ratio and nuclei roundness (Figure 2j4,k,m). However, FB nuclei in Novatach were significantly smaller compared to the FBs in CTRL (Figure 2l). There were no significant differences between the groups regarding the FB culture purity, FB proliferation rates, and FB viability (Figure 2n–p), demonstrating that all three hydrogels enabled normal FB survival and growth. Arrowheads in Figure 2j indicate THY1^+^/EdU^+^ proliferating FBs. All data can be found in Table S2.

Thus, all three hydrogels allowed cell adhesion and proliferation. Of note, only Cultrex BME induced an elongated morphology in SCs and FBs, rendering SCs similar to their distinct repair-phenotype.

### Cells in Cultrex BME Exhibited Oriented Collective Migration

After prolonged time without axon regeneration, the distal nerve stump and target tissue chronically denervate.^25^ Chronic denervation leads to SC atrophy,^28^ and the basal lamina and bands of Büngner degenerate before the regeneration process is completed.^29^ Time is therefore a crucial factor for functional recovery of long nerve defects, and cells must migrate efficiently to the injury site.^25^

**Figure 3:**
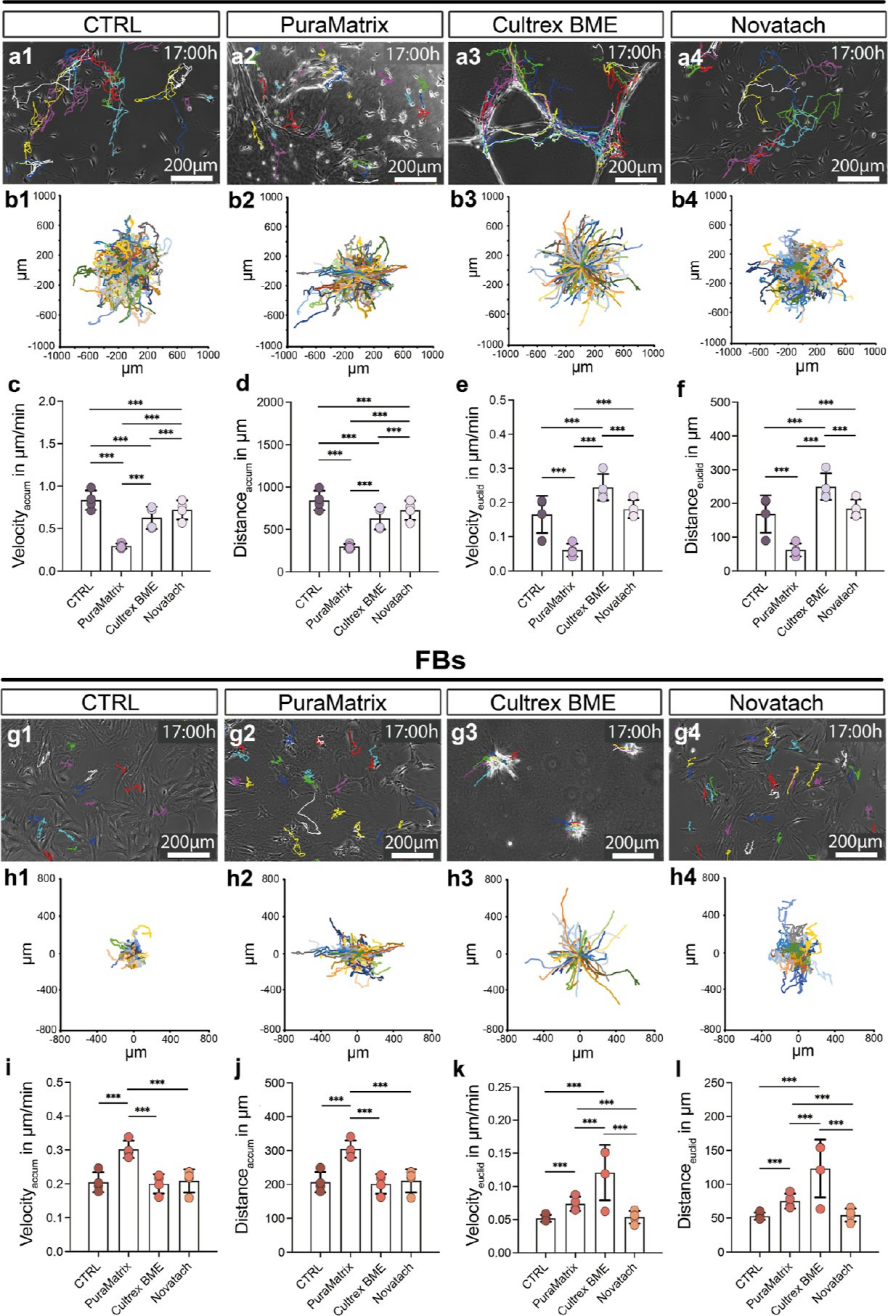
Evaluation of migratory potential of SCs and FBs in hydrogels vs CTRL. (a) Magnifications of representative images of SCs after 17 h live cell imaging. The colored lines each represent a SC’s migratory track. (b) Colored lines in the coordinate system represent each cell starting at 0 (center) for each condition. (c) Diagram depicts the mean ± SD accumulated velocity (velocity_accum_) of SCs in μm/min (*n* = 4). (d) Diagram depicts the mean ± SD accumulated (total) distance (distance_accum_) of SCs in μm (*n* = 4). (e) Diagram depicts the mean ± SD Euclidean (effective) velocity (velocity_euclid_) of SCs in μm/min (*n* = 4). (f) Diagram depicts the mean ± SD effective distance (distance_euclid_) of SCs in μm (*n* = 4). (g) Magnifications of representative images of FBs after 17 h of live cell imaging. The colored lines each represent a FB’s migratory tracks. (h) Colored line in the coordinate system represents each cell starting at 0 (center) for each condition. (i) Diagram depicts the mean ± SD velocity_accum_ of FBs in μm/min (*n* = 4). (j) Diagram depicts the mean ± SD distance_accum_ of FBs in μm (*n* = 4). (k) Diagram depicts the mean ± SD velocity_euclid_ of FBs in μm/min (*n* = 4). (l) Diagram depicts the mean ± SD distance_euclid_ of FBs in μm (*n* = 4). * *p*-value < 0.05, ** *p*-value < 0.01, *** *p*-value < 0.001.

To provide insights into the motility behavior of both SCs and FBs within the hydrogels, we performed live cell imaging. Every 10 min, an image was obtained over a period of 17 h. Figure 3a depicts the endpoint of the SC live cell imaging experiment after 17 h for all conditions. Each colored line represents the migratory path of one tracked SC throughout the 102 pictures. The coordinate systems in Figure 3b show the path of each cell starting at 0 (center). The SCs in the three hydrogels were significantly slower and covered less total distance compared to the CTRL. SCs in PuraMatrix were the slowest and covered the least distance in total, while the SCs in Novatach migrated slower than the SCs in CTRL but faster than the SCs in Cultrex BME (Figure 3c,d). Regarding the effective (Euclidean) velocity and distance covered by the cells, the SCs in Cultrex BME covered significantly more distance effectively and were faster than the SCs in the other conditions (Figure 3e,f). This finding indicates that Cultrex BME promotes directed migration of SCs.

Figure 3g shows the endpoint of the FB live cell imaging experiment after 17 h. The coordinate systems in Figure 3h show the path of each cell starting at 0 (center). FBs were significantly faster and covered more total distance in PuraMatrix compared to the other conditions (Figure 3i,j). Similar to the SCs in the hydrogels, we saw an impact on the effective velocity and distance covered by the FBs (Figure 3k,l). FBs covered the least distance effectively in CTRL and in Novatach, while the FBs in Cultrex BME covered significantly more distance effectively and were faster effectively compared to the FBs in the other conditions (Figure 3k,l). All data can be found in Table S2.

### Laminin in Cultrex BME but Not Collagen Plays a Role in Oriented Cell Migration

To this point, we identified Cultrex BME as a promotor of elongated cells, as well as oriented, collective migration in both SCs and FBs. The basement membrane hydrogel consists of two main components; laminin I and rat tail collagen I. To investigate whether one or both of these two components are responsible for the increased directed migration, we next examined two additional hydrogels made of these components, Cultrex 3D Cell Culture Matrix Laminin I (Cultrex Laminin) and Cultrex 3D Cell Culture Matrix Rat Tail Collagen I (Cultrex Collagen).

**Figure 4:**
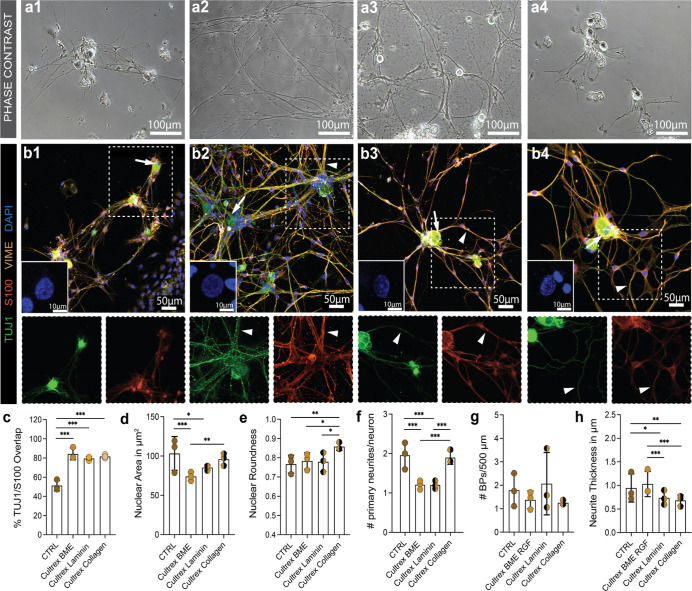
DRG neuron seeded in three Cultrex hydrogels and control. (a) Phase contrast micrographs of DRG neuron cultures on (a1) PDL/laminin-coated cell culture dishes (CTRL), (a2) Cultrex BME, (a3) Cultrex Laminin, and (a4) Cultrex Collagen. (b) Representative confocal micrographs of cultures stained for TUJ1 in green, S100 in red-brown, vimentin (VIME) in orange, and DAPI in blue. Images in the left corner are representative micrographs of a DAPI^+^ cell nucleus of a single DRG neuron, pointed out by the white arrows. White arrowheads show SC/neurite alignment. (c) Diagram depicts the mean ± SD percentage of S100 (SC) and TUJ1 (DRG neurons) overlap (*n* = 3). (d) Diagram depicts the mean ± SD nuclei size of DRG neurons in μm^2^ (*n* = 3). (e) Diagram depicts the mean ± SD nuclei roundness (*n* = 3). (f) Diagram depicts the mean ± SD number of primary neurites (*n* = 3). (g) Diagram depicts the mean ± SD number of BPs per 500 μm (*n* = 3). (h) Diagram depicts the mean ± SD neurite thickness in μm (*n* = 3). * *p*-value < 0.05, ** *p*-value < 0.01, *** *p*-value < 0.001.

First, we seeded DRG cultures into the two new Cultrex hydrogels and again in Cultrex BME (Figure 4a). It is worth noting that a higher SC/neurite alignment was observed in all three hydrogels compared to PDL/laminin (CTRL) (Figure 4c, pointed out by arrowheads in Figure 4b). The DRG neurons’ nuclei were smallest in Cultrex BME (Figure 4d), while they were roundest in Cultrex Collagen (Figure 4e). In both Cultrex BME and Cultrex Laminin, the neurons mostly had only one primary neurite (Figure 4f). Furthermore, while there was no difference in the number of BPs between the four conditions (Figure 4g), we observed thinner neurites in both Cultrex Laminin and Cultrex Collagen compared to Cultrex BME and CTRL (Figure 4h). In summary, all three Cultrex hydrogels induced SC/neurite alignment, but only Cultrex BME and Cultrex Laminin promoted the growth of a single primary neurite. All data can be found in Table S2.

**Figure 5:**
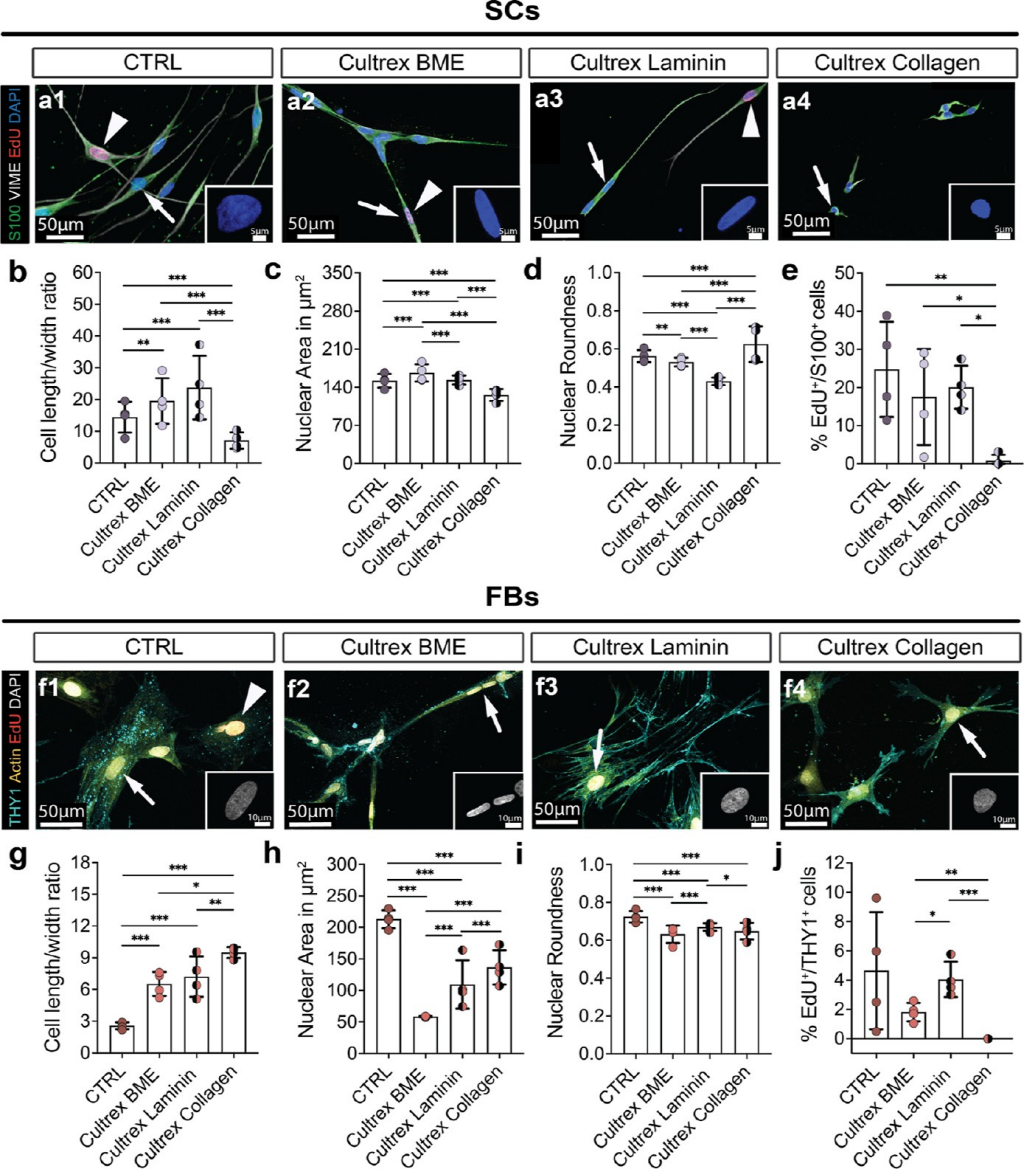
Morphological analyses of SCs and FBs seeded on three Cultrex hydrogels and in CTRL. (a) Representative confocal micrographs of SC cultures on (a1) PLL/laminin coated cell culture dishes (CTRL), (a2) Cultrex BME, (a3) Cultrex Laminin, and (a4) Cultrex Collagen stained for S100 in green, vimentin (VIME) in gray, EdU in red, and DAPI in blue. Small images in the right corner are representative micrographs of a DAPI^+^ cell nucleus of a single SC, pointed out by the white arrows. (b) Diagram depicts the mean ± SD length/width ratio of SCs on PLL/laminin (CTRL) and hydrogels (*n* = 4). (c) Diagram depicts the mean ± SD nuclei size of SCs in μm^2^ for the four groups (*n* = 4). (d) Diagram depicts the roundness of SC nuclei on PLL/laminin and hydrogels ± SD (*n* = 4). (e) Diagram shows the mean ± SD percentage of S100^+^/EdU^+^ cells (proliferating SCs) for each group (*n* = 4). (f) Representative confocal micrographs of FB cultures on (f1) uncoated cell culture wells (CTRL), (f2) Cultrex BME, (f3) Cultrex Laminin, and (f4) Cultrex Collagen stained for THY1 in cyan, actin in gold, EdU in red, and DAPI in gray. Small images in the right corner of the image are representative micrographs of a DAPI^+^ cell nucleus of a single FB, pointed out by the white arrows. (g) Diagram depicts the mean ± SD length/width ratio of FBs in control and hydrogels (*n* = 4). (h) Diagram depicts the mean ± SD nuclei size of FBs in μm^2^ (*n* = 4). (i) Diagram depicts the mean ± SD roundness of FB nuclei in control and hydrogels (*n* = 4). (j) Diagram depicts the mean ± SD number of THY1^+^/EdU^+^ FBs (*n* = 4). * *p*-value < 0.05, ** *p*-value < 0.01, *** *p*-value < 0.001.

Next, we again assessed the effects of the hydrogels on SC and FB morphology. In Cultrex Laminin, the SCs were significantly more elongated compared to SCs on PLL/laminin (CTRL) and Cultrex Collagen, but showed no significant difference to Cultrex BME (Figure 5a3,b). Moreover, the SC nuclei in Cultrex Laminin were the least round, significantly differing from all other conditions (Figure 5a3,c,d). Phase contrast micrographs of SCs in Cultrex Collagen showed a rather round morphology with short processes (Figure 5a4). This was confirmed by immunofluorescence stainings, which demonstrated a significantly lower length/width ratio of SCs in Cultrex Collagen compared to the other groups (Figure 5b). Moreover, their nuclei were significantly smaller and rounder (Figure 5a4,c,d). Lastly, to complete all morphological assessments from the first experiments, we compared the proliferation rates of the cells in Cultrex Collagen and Cultrex Laminin. While there were no differences between the proliferation rates of SCs in control, Cultrex BME, and Cultrex Laminin, the SCs in Cultrex Collagen were almost non-proliferating, significantly differing from the other conditions (Figure 5e).

FB cultures were stained for FB-marker THY1 and proliferation marker EdU in combination with actin as a marker for all cells. The FBs were more elongated in Cultrex BME and Cultrex Laminin compared to the FBs on uncoated wells (CTRL), but less elongated to the FBs in Cultrex Collagen, in which the cells were most elongated (Figure 5f,g). The FBs nuclei were biggest and roundest on uncoated wells, significantly decreasing in size from FBs in Cultrex Collagen via Cultrex Laminin to FBs in Cultrex BME (Figure 5h). The nuclei were least round in Cultrex BME (Figure 5i). Lastly, FBs did not proliferate at all in Cultrex Collagen, but proliferated significantly higher in Cultrex Laminin compared to Cultrex BME and Cultrex Collagen (Figure 5j).

**Figure 6:**
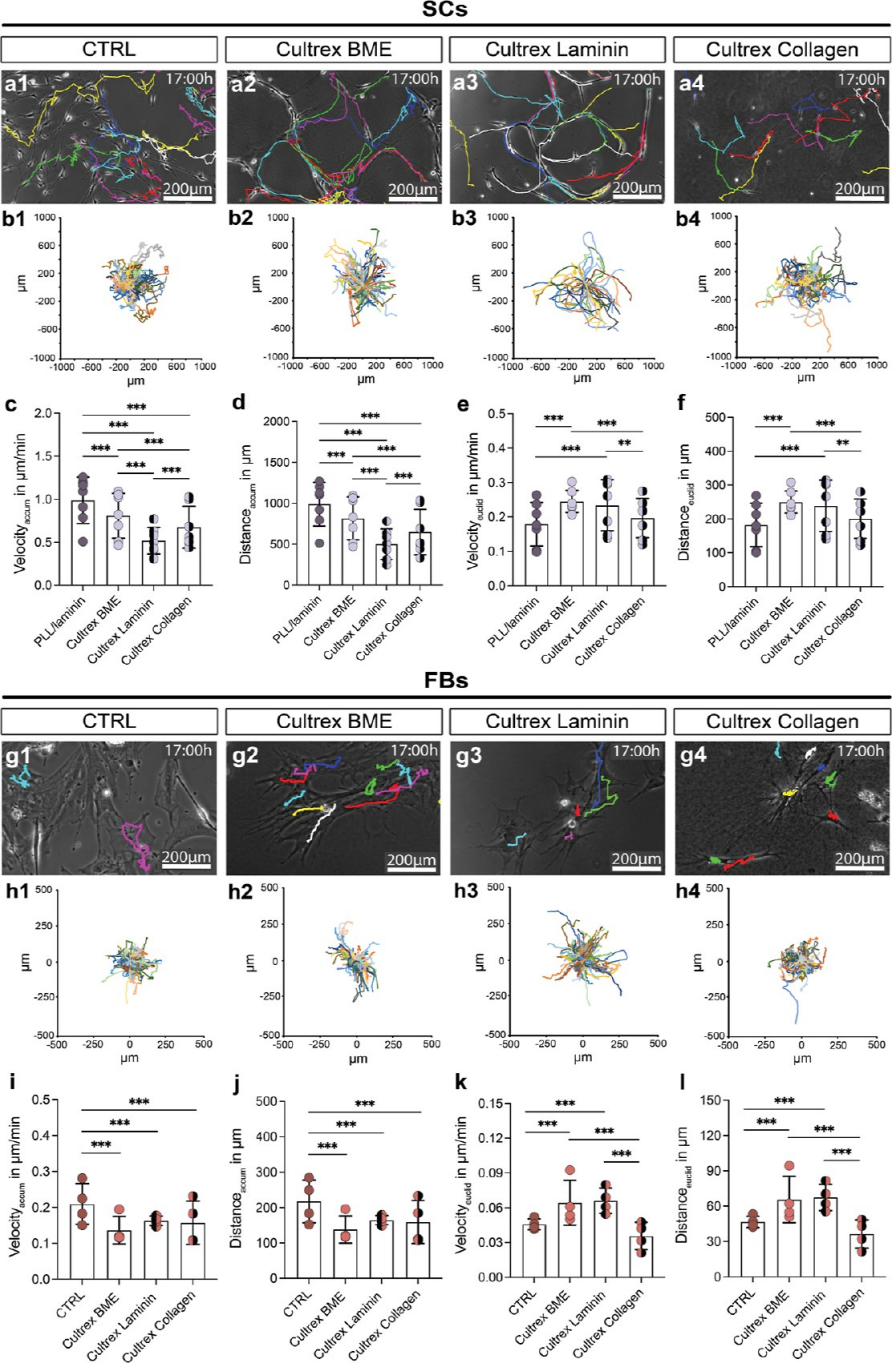
Live cell imaging of SCs and FBs seeded on three Cultrex hydrogels and in control. (a) Magnifications of representative images of SCs on (a1) PLL/laminin-coated cell culture dishes (CTRL), (a2) Cultrex BME, (a3) Cultrex Laminin, and (a4) Cultrex Collagen after 17 h. The colored lines each represent an SC’s migratory tracks. (b) Colored line in the coordinate system represents each cell starting at 0 (center) for each condition. (c) Diagram depicts the mean ± SD accumulated velocity (velocity_accum_) of SCs in μm/min (*n* = 6). (d) Diagram depicts the mean ± SD accumulated (total) distance (distance_accum_) of SCs in μm (*n* = 6). (e) Diagram depicts the mean ± SD effective velocity (velocity_euclid_) of SCs in μm/min (*n* = 6). (f) Diagram depicts the mean ± SD effective distance (distance_euclid_) of SCs in μm (*n* = 6). (g) Magnifications of representative images of FBs on (f1) uncoated cell culture dishes (CTRL), (f2) Cultrex BME, (f3) Cultrex Laminin, and (f4) Cultrex Collagen after 17 h. The colored lines each represent a FB’s migratory tracks. (h) Colored line in the coordinate system represents each cell starting at 0 (center) for each condition. (i) Diagram depicts the mean ± SD accumulated velocity (velocity_accum_) of FBs in μm/min (*n* = 4). (j) Diagram depicts the mean ± SD accumulated (total) distance (distance_accum_) of FBs in μm (*n* = 4). (k) Diagram depicts the mean ± SD effective velocity (velocity_euclid_) of FBs in μm/min (*n* = 4). (l) Diagram depicts the mean ± SD effective distance (distance_euclid_) of FBs in μm (*n* = 4). The bar represents the mean of all donors; * *p*-value < 0.05, ** *p*-value < 0.01, *** *p*-value < 0.001.

Live cell imaging of SCs in the three Cultrex hydrogels showed that the migratory path for each SC appears straighter in both Cultrex BME and in Cultrex Laminin compared to Cultrex Collagen (Figure 6a,b). In Cultrex BME, SCs were significantly slower and covered less distance than in CTRL, followed by the cells in Cultrex Collagen (Figure 6c,d). The SCs were slowest and covered the least total distance in Cultrex Laminin (Figure 6c,d). Looking at the effective velocity and distance, SCs in Cultrex BME and in Cultrex Laminin covered the most distance and were the fastest, significantly differing from both SCs in control and SCs in Cultrex Collagen (Figure 6e,f). The FBs were significantly slower and covered less distance in total in all three hydrogels compared to the FBs on uncoated wells (CTRL) (Figure 6g–j). However, their effective velocity and covered distance was significantly higher in both Cultrex BME and Cultrex Laminin compared to control and Cultrex Collagen (Figure 6k,l). All data can be found in Table S2.

To summarize, both SCs and FBs showed an increase in directionality in Cultrex BME and Cultrex Laminin but not in Cultrex Collagen. These results indicate that laminin but not collagen is the driver of the directed cell migration seen in Cultrex BME.

### Increased Concentration of Laminin Correlates with Cell Elongation but Does Not Affect Directed Cell Migration Equivalently

**Figure 7:**
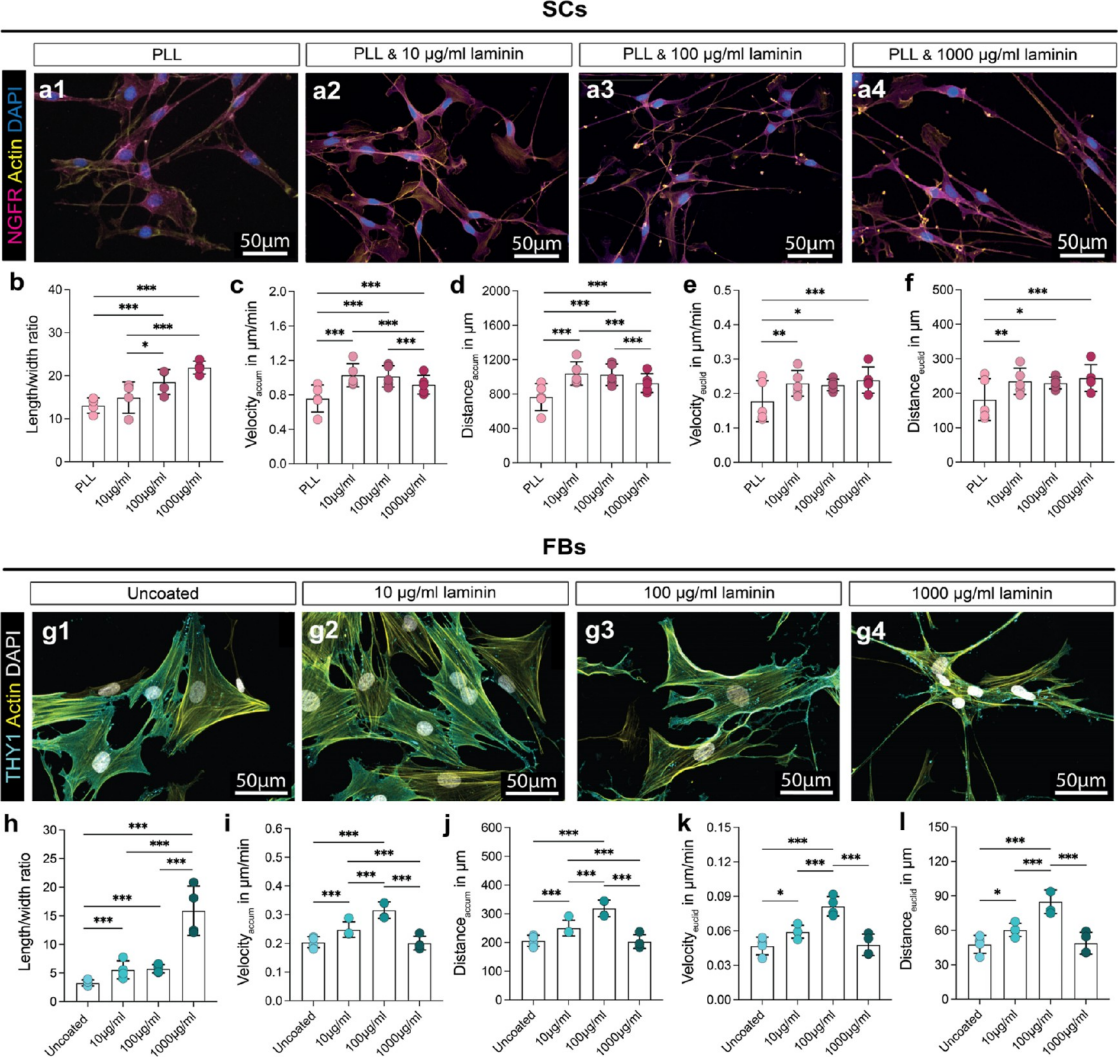
SCs and FBs seeded on increasing laminin concentration. (a) Representative confocal micrographs of SCs stained for NGFR in magenta, Actin in yellow, and DAPI in blue on (1) PLL and PLL/laminin coating with (2) 10, (3) 100, and (4) 1000 μg/mL laminin concentration. (b) Diagram depicts the mean ± SD length to width ratio (*n* = 5). (c) Diagram depicts the mean ± SD velocity_accum_ in μm/min (*n* = 5). (d) Diagram depicts the mean ± SD distance_accum_ in μm (*n* = 5). (e) Diagram depicts the mean ± SD velocity_euclid_ in μm/min (*n* = 5). (f) Diagram depicts the mean ± SD distance_euclid_ in μm (*n* = 5). (g) Representative confocal micrographs of FBs stained for THY1 in cyan, actin in yellow, and DAPI in white on (1) uncoated wells and laminin coating with (2) 10, (3) 100, and (4) 1000 μg/mL laminin concentration. (h) Diagram depicts the mean ± SD length to width ratio (*n* = 4). (i) Diagram depicts the mean ± SD velocity_accum_ in μm/min (*n* = 4). (j) Diagram depicts the mean ± SD distance_accum_ in μm (*n* = 4). (k) Diagram depicts the mean ± SD velocity_euclid_ in μm/min (*n* = 4). (l) Diagram depicts the mean ± SD distance_euclid_ in μm (*n* = 4); * *p*-value < 0.05, ** *p*-value < 0.01, *** *p*-value < 0.001.

So far, our experiments have revealed that laminin but not collagen in Cultrex BME plays a role in enhancing directed migration in the cells. To further verify whether laminin can be associated with increased effective velocity, we next investigated the migratory behavior of SCs and FBs on PLL coatings with increasing laminin concentration ranging from 10 to 1000 μg/mL (Figure 7). PLL pre-coating is necessary as positively charged poly-electrolytes like PLL enhance attachment and process formation of negatively-charged SCs (Figure S1).^26^ Therefore, SCs were seeded on a PLL/laminin coating with increasing laminin concentration and as controls, on the standard PLL/laminin coating as well as a PLL coating without laminin (Figure 7a–g). As the SCs on the standard PLL/4.8 μg/mL laminin coating behaved similar to the 10 μg/mL laminin coating, we have excluded these results for simplicity and better comparison from Figure 7, but they can be found in Figure S2. As FBs are physiologically plastic-adherent, FBs were seeded on uncoated wells as control and on a laminin coating with increasing laminin concentration (Figure 7g–l).

SCs seeded on 1000 μg/mL laminin had a significantly higher length to width ratio than SCs seeded on 10 μg/mL laminin, but there were no differences in elongation between SCs on PLL and 10 μg/mL laminin (Figure 7b). Live cell imaging videos revealed a general effect of laminin on migratory behavior of SCs as SCs seeded on laminin were significantly faster and covered more distance than the SCs on PLL only. However, with the increasing laminin concentration, the SCs’ velocity first increased with significant changes and later decreased at a concentration of 1000 μg/mL (Figure 7c,d). SCs seeded on PLL also had a significantly slower effective velocity and distance covered than SCs seeded on PLL, with laminin indicating an effect of laminin on directed migration, but we did not see any differences between the effective velocity and effective distance covered by the cells in the three laminin concentrations (Figure 7e,f). The coordinate systems of the live cell imaging data can be found in Figure S3.

The FBs seeded on laminin were significantly more elongated than on uncoated wells and showed a significant increase in elongation on 1000 μg/mL laminin (Figure 7h). Live cell imaging videos revealed that the FBs’ velocity and distance covered (total as well as effective) significantly increased from uncoated wells to wells coated with 10 μg/mL and additionally with 100 μg/mL laminin, but declined to values comparable to control on 1000 μg/mL laminin (Figure 7i–l).

Hence, while the increasing laminin concentrations promoted cell elongation, the experiments did not reveal such a correlation between elongation and directed migration. Indeed, we observed that laminin increased the directed migration of both SCs and FBs; however, directionality of cells decreased at a concentration of 1000 μg/mL laminin. Thus, laminin cannot be the sole driver behind directed migration. The coordinate systems of the live cell imaging data can be found in Figure S3.

### Cultrex BME and Cultrex Laminin Exhibit Distinct Mechanical Properties

Damage to tissue entails structural changes to the ECM to which cells need to adapt. The accrued scars after PNI, for example, represent a much stiffer tissue than the healthy ECM and can even act as a barrier for regenerating neurons.^30^ Our results so far revealed that laminin promotes cell elongation but is not solely responsible for the increased directed migration. However, the three-dimensional matrix of the hydrogels, the incorporated laminin, as well as its inducing mechanical properties could be drivers for the oriented, collective migration in both SCs and FBs. We therefore continued with detailed material analyses of the hydrogels in our experiments.

First, we conducted SEM of SCs and FBs on the various PLL/laminin coatings and their controls but did not find any visible topographical differences in surface structures. The micrographs can be found in Figure S4.

Next, we examined the three-dimensional matrices. While preparing the hydrogels based on the manufacturers’ recommendations, variations in the gels’ morphology could be observed. While all three Cultrex hydrogels formed a thick three-dimensional matrix, Novatach appeared as a thin layer similar to a coating.

**Figure 8:**
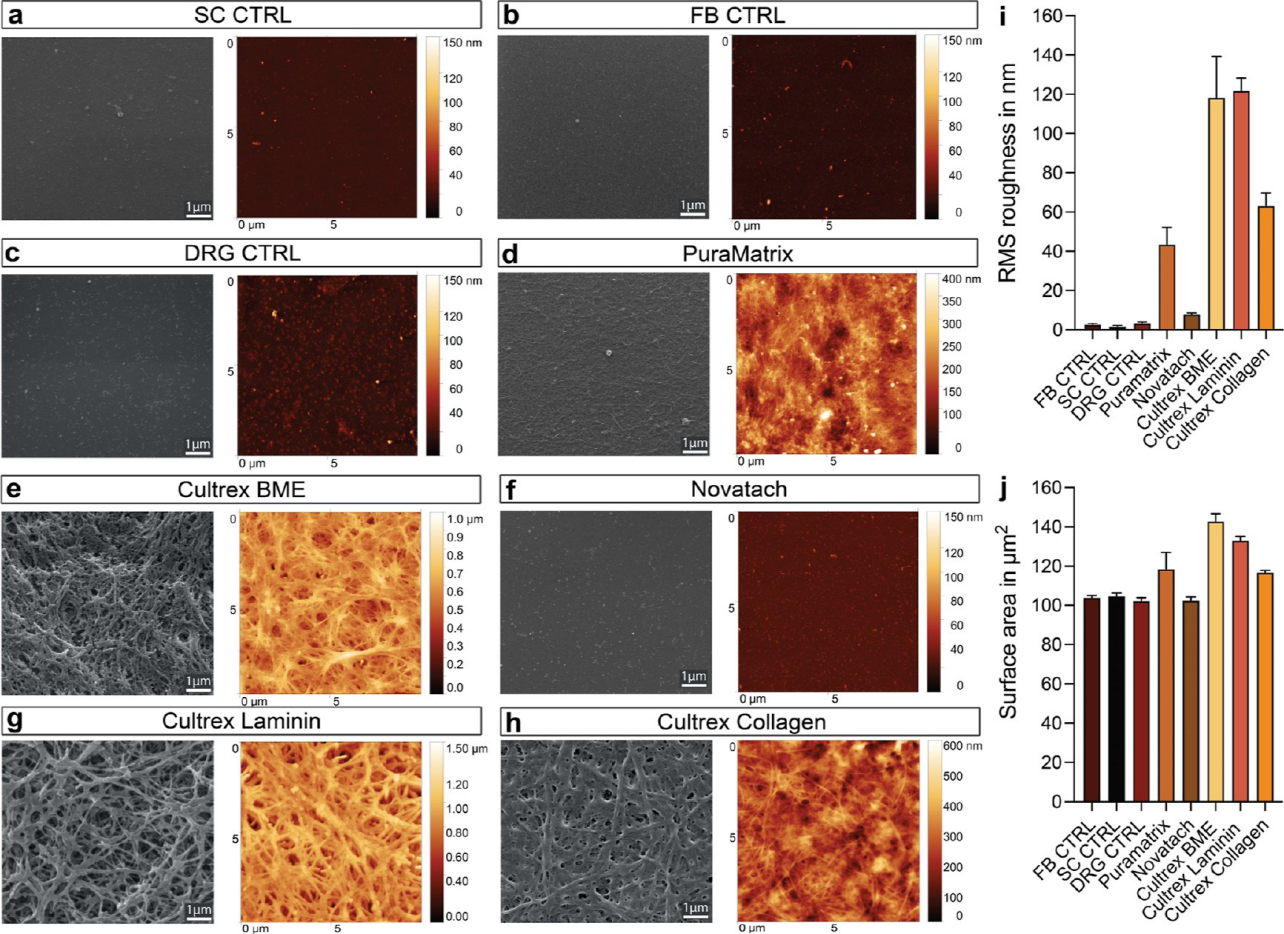
Evaluation of surface topography of hydrogels. SEM visualized smoother surface topography for (a–c left images) control coatings (PLL/laminin for SCs, uncoated for FBs, and PDL/laminin for DRGs) and (f, left image) Novatach in comparison to the features visible on (d, left image) PuraMatrix, (e, left image) Cultrex BME, and (g,h, left images) Cultrex Laminin and Cultrex Collagen. (a–e) Atomic force micrographs (a–h, right images) visualized differences in (i) surface roughness and (j) surface area between the hydrogels. (i) Diagram depicts the mean RMS roughness in nm for the control coatings and each hydrogel. (j) Diagram depicts the mean surface area for the control coatings and each hydrogel. Data are presented as mean ± SD from three technical replicates.

SEM and AFM were employed to quantify the differences between the morphology of gels and control coatings (PLL/4.8 μg/mL laminin, PDL/laminin, and uncoated well) (Figure 8a–h). Our phase contrast micrographs revealed that the controls and Novatach appeared smooth with a mostly homogenous surface (Figure 8a–c,f). PuraMatrix and the three Cultrex hydrogels on the other hand seemed to have more surface features (Figure 8d,e,g,h) with Cultrex BME and Cultrex Laminin seemingly having the roughest surfaces, appearing almost like a meshwork of fibers with many pores. Analysis of AFM micrographs confirmed these observations. The control coatings had a smooth surface with a root mean square (RMS) roughness below 4 nm (Figure 8i). Novatach had a slightly higher mean RMS roughness than the CTRLs, while PuraMatrix, followed by Cultrex Collagen (Figure 8i), showed a significantly higher surface roughness. Cultrex BME and Cultrex Laminin had the highest mean RMS roughness, almost double compared to PuraMatrix and Cultrex Collagen (Figure 8i). As rougher surfaces should offer more surface area, we further quantified the surface area from the AFM measurements over an area of 100 μm^2^ of the hydrogels. Indeed, Cultrex BME and Cultrex Laminin had the highest surface areas followed by PuraMatrix and Cultrex Collagen (Figure 8j). There were no significant differences between Novatach and controls, which all had a mean surface area of around 103 μm^2^. Thus, while Cultrex BME, Cultrex Laminin, PuraMatrix, and Cultrex Collagen show a certain surface roughness and area expected from hydrogels, Novatach exhibited a similar topography as the coatings. All data can be found in Table S2.

**Figure 9:**
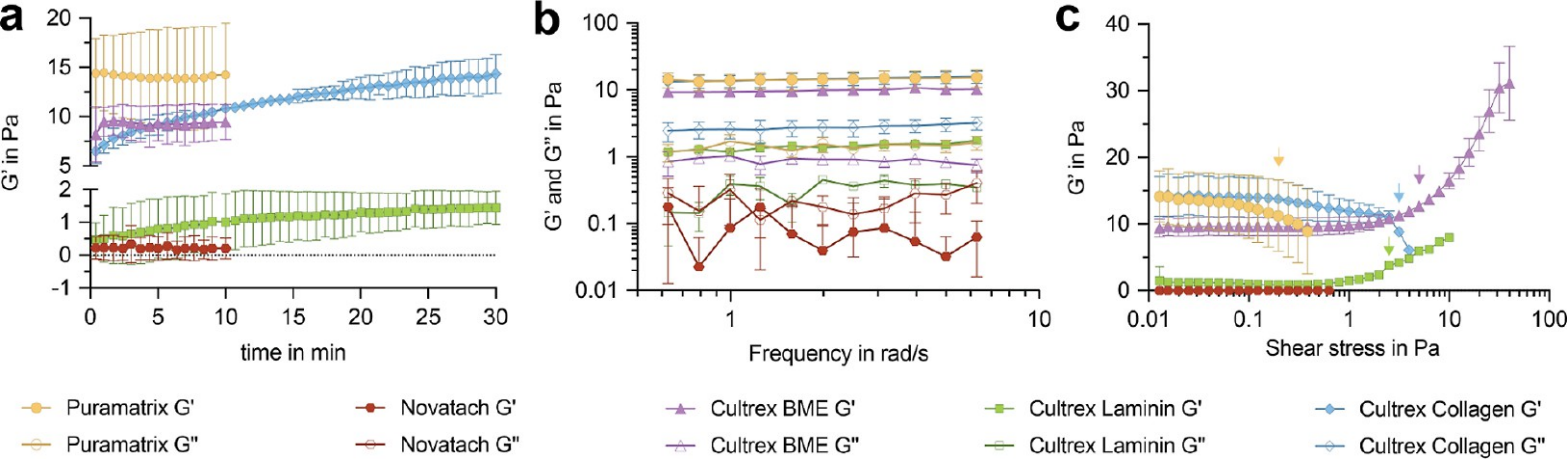
Rheological analysis of hydrogels. (a) Diagram depicts *G*′ of the five hydrogels within 30 min. (b) Diagram depicts changes in *G*′ and *G*″ of the five hydrogels with increasing frequency. (c) Diagram depicts changes in *G*′ with increasing shear stress. Arrows indicate yield points (points at which *G*′ changed more than 5%). Data are presented as mean ± SD from three technical replicates.

In the next step, rheological analyses of the five hydrogels were performed. These experiments allowed us to test their stiffness and plasticity. To generate and equilibrate the gels in the rheometer gap, their stiffness (expressed as storage modulus, *G*′) was recorded for 30 min (also according to manufacturers’ instructions). *G*′ of both PuraMatrix and Novatach was constant within the stabilization period, indicating minimal stress relaxation due to preceding manipulations (Figure 9a). In contrast, the *G*′ of Cultrex BME decreased slightly in the beginning of the time sweep, but remained constant afterwards indicating that the sample needed some time to relax following pipetting and closing the gap (Figure 9a). The *G*′ of Cultrex Laminin and Cultrex Collagen steadily increased, with Cultrex Laminin stabilizing after about 25 min. Cultrex Collagen continuously gained elasticity, but we stopped the time sweep after 30 min to stay consistent with the time prior to cell seeding as recommended by the manufacturer. There was high variation in stiffness between the five hydrogels in particular between Cultrex Laminin and Cultrex BME (Figure 9a).

The subsequent frequency sweep tests showed the gelatinous nature (*G*′ > *G*″) of all hydrogels except Novatach (Figure 9b). PuraMatrix and the three Cultrex hydrogels remained in their linear states throughout the test and oscillated as a whole in the gap. In contrast, Novatach had very weak and unstable *G*′ values in this frequency range and predominantly flew in the gap (*G*″ > *G*′) (Figure 9b).

Following these steady state tests, the hydrogels were subjected to higher sinusoidal shear stress amplitudes to obtain their yielding behavior (Figure 9c). The yield points as indicated by the arrow in Figure 9c depict the shear stress at which the material leaves its equilibrium state. Novatach was unstable right from the beginning of the test and became fully fluidic at around 0.6 Pa (*G*′ became zero). The shear moduli of PuraMatrix remained stable up to 0.20 Pa (yield point), but then dropped until the gel became fully fluidic at 0.40 Pa (Figure 9c). Cultrex Collagen’s shear moduli steadily decreased until it yielded at 8.85 Pa (Figure 9c), while Cultrex BME yielded at a shear stress of around 12.79 Pa and Cultrex Laminin at 3.83 Pa (Figure 9c). In contrast to the other hydrogels, Cultrex BME and Cultrex Laminin showed a stress-stiffening behavior. This means that the laminin-containing gels did not become progressively weaker with the shear strain, but instead became stiffer with higher loads. In total, both hydrogels demonstrated a strain between 85.00% and 90.00% (Cultrex BME: 89.93% and Cultrex Laminin: 87.03%). As only the laminin containing hydrogels showed an increase in cell directionality (compare with Figure 6), the rheological results indicate a possible synergy between laminin and the mechanical properties of hydrogels and cell directionality.

## Discussion

Biomaterials like hydrogels play a central role in facilitating regeneration by providing structural support in the form of an artificial ECM.^31^ Among others, they have found their use in PNR as a filling material for conduits,^8^ where the applied hydrogel needs to favorably interact with the cellular components surrounding the injury site and, ideally, enhance cellular properties necessary for PNR.^16^ The search for the ideal biomaterial in peripheral nerve reconstruction is ongoing, and a variety of substances from various pharmaceutical companies are commercially available.^32^

For this reason, this project systematically compared three hydrogels advertised for PNR, namely, the synthetic, polypeptide hydrogel PuraMatrix, the basement membrane extract hydrogel Cultrex BME, and the RDG-coupled alginate hydrogel Novatach and investigated their effect on SCs, fetal bovine serum, and DRG neurons *in vitro*. After we identified Cultrex BME as a promotor of elongated cells, as well as oriented, collective migration in both SCs and FBs, detailed follow-up experiments were conducted. To investigate the reasons and to identify the key component behind Cultrex BME’s effect on the cells, we repeated the experiments with Cultrex Laminin, a hydrogel consisting solely of laminin and Cultrex Collagen, a hydrogel consisting solely of collagen. Lastly, using state-of-the-art methods, the hydrogels’ material properties were analyzed.

The success of PNR upon transection is the target organ re-innervation of the re-growing axons, a process that involves the coordinated action of SCs and FBs at the injury site.^33^ Hence, hydrogels used for PNR must encourage SC/neurite alignment to direct neurite outgrowth. In PuraMatrix, the DRG neurons had thicker neurites and more branching points. However, in Cultrex BME and Cultrex Laminin, the DRG neurons mostly formed one primary neurite with less branching points, encouraging fast and directed sprouting of axons in PNR.^34^ It has been demonstrated that developing neurons extend several immature neurites, of which one persists and becomes the axon, while the others become dendrites.^35^ Wissner-Gross et al.^36^ confirmed these findings and in addition reported that the longer a neurite became the faster it grew, demonstrating that neurite growth was accelerative. This suggests that a decreased number of neurites per cell is of advantage for regenerating neurons because the neuron is concentrating its resources on fewer neurites and consequently growing faster. Our results further demonstrated an increased SC/neurite alignment in all three Cultrex hydrogels. Endo et al.^37^ cultured DRG neurons with SCs in two conditions (with and without direct contact) and reported that direct contact with SCs significantly increased neurite outgrowth of neurons. This, in combination with the fact that SCs in Cultrex BME and Cultrex Laminin formed elongated and net-like structures, suggested that those two hydrogels might facilitate the formation of bands of Büngner necessary for PNR.^38^

The cells surrounding the injury site undergo specific adaptations in response to PNI. For instance, the SCs transdifferentiate into a repair state that is characterized by a specific, more elongated phenotype.^18^ The distinct morphologies of SCs in Cultrex BME and Cultrex Laminin are remarkable as it highly resembles the elongated SC phenotype necessary for PNR. Similar, the FBs in our experiment exhibited a more elongated phenotype in the Cultrex hydrogels. Past studies correlated a more elongated FB appearance with rougher surfaces,^39^ and it is hypothesized that this effect depends on the increased surface area of rough substrates.^39^ It has been suggested that the pores and filaments of rough substrates offer cells supporting elements to which they attach.^40^ Interestingly, although these results support our experiment results as SCs were significantly more elongated in the hydrogels with the roughest surface, this was not true for the FBs. Moreover, in comparison to the hydrogels, the controls had the smoothest surface, but neither the SCs nor the FBs had the lowest length/width ratio in the controls. The laminin concentration experiments further revealed that even though there was no difference in surface structure, SCs and FBs were more elongated on the highest laminin concentration. Hence, our findings revealed that while there is no correlation between surface structure and cell spreading, laminin seems to be an important driver behind cell elongation and its exact role should be investigated in further experiments. A recent publication by Xue et al.^41^ compared a decellularized peripheral nerve matrix to a collagen hydrogel and also found that laminin promoted SC spreading and decreased cell circularity. Lin and Bertics^42^ demonstrated that FBs bound to laminin are more elongated and that these cells possess high-affinity epidermal growth factor binding. Moreover, their study revealed that ∝6-containing integrins play a role in this laminin-dependent attachment. They hypothesized that the altered cell morphology on laminin may be due to changes in cytoskeletal organization, as laminin, laminin-binding proteins, and integrins are all associated with the cytoskeleton.^42^

Hydrogels in PNR should allow for cell division as the repair phenotype of SCs is characterized by an initial increase in cell proliferation.^43^ Our results revealed that while there were no significant differences between the other conditions, the SCs in Cultrex BME and Cultrex Collagen proliferated significantly less. This contrasts findings from Soucy et al.^44^ who reported an increase in proliferation of SCs that were encapsulated in a collagen-based hydrogel. Our findings are in accordance with results from Yoshino et al.^45^ who cultured SCs in Matrigel, a basement membrane hydrogel of similar origin as Cultrex BME. They noted decreased proliferation rates compared to SCs on uncoated culture dishes. Although PNI causes a burst of SC proliferation,^43^ the targeted inhibition of SCs proliferation had no effect on the regenerative outcome in *in vivo* experiments.^46^ This indicates that, against common views, SC proliferation may not play a fundamental role in PNR.^47^ The authors hypothesized that the increased SC proliferation rates after injury might rather be a strategy to ensure sufficient cell numbers by generation of excess cells. These cells are then removed by induction of apoptosis.^47^ Thus, a lower proliferation rate of SCs as seen in Cultrex BME might not present as a hindrance in PNR after PNI.

The most noteworthy finding of our study is that the cells exhibited directed migration in Cultrex BME and Cultrex Laminin. Directed movement of SCs could potentially be a game changer in peripheral nerve repair^48^ as preventing SC migration has been shown to markedly reduce regeneration.^49^ A coordinated interaction between SCs and FBs is important to bridge the gap between the distal and proximal nerve stump and guide axonal re-growth.^33^ As time is a critical factor after nerve injury, directed migration of SCs could enable faster regeneration and thereby significantly increase the possibility of successful recovery.^50^ To determine which components of Cultrex BME are responsible for our findings, we seeded SCs and FB cultures in Cultrex Laminin consisting solely of laminin I and Cultrex Collagen consisting exclusively of rat tail collagen I. These experiments showed that, while there was no significant difference between both SCs and FBs in Cultrex Collagen and the control, the cells exhibited oriented, collective migration in both, Cultrex BME and Cultrex Laminin. To verify if laminin is the sole driver behind directed migration, we seeded SCs and FBs on increasing laminin concentrations and found that from 10 to 1000 μg/mL laminin, the cells exhibited a more elongated morphology. The presence of laminin also promoted the directionality of cells, but there was no correlation between the various laminin concentration and cell migration. Thus, laminin alone cannot induce the oriented migratory behavior in SCs and FBs seen in the laminin containing hydrogels.

To elucidate the possible reasons behind the oriented, collective migration of cells in Cultrex BME and Cultrex Laminin, we performed material analyses of the hydrogels. We did not find correlations in the stiffness moduli of the hydrogels. However, the most striking difference between the hydrogels is the ability of Cultrex BME and Cultrex Laminin to stiffen when exposed to shear deformation. Strain- or stress-stiffening is a characteristic of many biological materials.^51^ It allows the deformed material to take up higher loads. The material’s architecture conforms to the strain and thereby gains a certain plasticity that can prevent fatal deformations of tissue in the body.^51^ Neurofilament networks, for example, can be strained over 400% until breakage (from *G*′ = 2.5 to 30 Pa).^51^ This non-linear elasticity is due to crosslinked, semiflexible filamentous proteins and is dependent on the contour length of the filaments and on the forces between the structural branching points.^51^ Analysis of the AFM images showed a more porous surface as well as a more fibrous nature of Cultrex BME and Cultrex Laminin compared to the other hydrogels, and even to Cultrex Collagen, which consists of thinner fibers that are more embedded into the matrix and therefore has shallower pores. Gnavi et al.^52^ cultured SCs on gelatin fibers of different diameters and showed that SC elongation and migration is indeed influenced by the fiber diameter. They correlated these findings with the SCs having fewer focal adhesion points and enhanced filopodia formation on thicker fibers.^52^ In concordance with that, the SCs in Cultrex BME and Cultrex Laminin might have used the gel’s fibers to migrate through the matrix and the mechanism behind this should be investigated in future experiments by possibly arranging the fibers. This might also be a reason for the collective, aligned migration that was especially visible in the SC cultures. Moreover, the larger pores visible in Cultrex BME and Cultrex Laminin more closely resemble the native ECM which is intrinsically microporous with pores between 2 and 30 μm.^53^ Hence, the porosity and fiber characteristics of Cultrex BME and Cultrex Laminin offer favorable properties for cell migration and should be considered in future studies.

All this suggests that SC and FB migration is fine-tuned by a complex interplay of fiber diameter, pore size, and laminin binding sites. Interestingly, Dent et al.^54^ demonstrated that laminin promotes the formation of filopodia-like protrusions in neurons. In turn, filopodia in a three-dimensional setting has been shown to sense the topography and stiffness of the matrix.^55^ Furthermore, enhanced filopodia formation in SCs has been correlated to an increase in cell elongation and migration.^52^ Thus, we propose that a combination of laminin and the mechanical properties of Cultrex BME and Cultrex Laminin promoted filopodia formation responsible for the directed migration of SCs and FBs. This should be addressed in further experiments such as super-resolution images of cells on coatings with various laminin concentrations as well as in hydrogels with various fiber diameters ideally with the same concentration of laminin. Comparison of hydrogels with varying fiber diameter and laminin concentration could also be of interest. In addition, the correlation between strain-stiffening with a porous and fibrous nature of hydrogels should be investigated. Moreover, it is necessary to elucidate the reasons underlying the not only directed but also collective migration of the SCs especially. The bands of Büngner formation in the SCs promoted by Cultrex BME and Cultrex Laminin could explain this phenomenon, as well as why we do not see aligned migration in the FB cultures. Moreover, the SCs who are intrinsically smaller than the FBs^56^ might have been able to migrate along the gels’ fibers, thereby appearing to move collectively. Another explanation might be that the hydrogels elicit the SCs to send chemotactic cues to follow each other. Lastly, one cell might create a “tunnel” within the hydrogel which other cells then use as well. A study by Lee et al.,^57^ for example, used two-photon laser scanning photolithography to guide the migration of FBs by creating channels through immobilized biomolecules. It would be interesting to investigate this by creating channels of the same size through the hydrogel.

A limitation of our study is the inexact protein concentrations of the used hydrogels. It is known that higher protein concentrations affect matrix stiffness and porosity.^58^ Cultrex BME’s major components include laminin, collagen IV, entactin/nidogen, and heparin sulfate proteoglycans at a protein concentration of 8–12 mg/mL. Cultrex Laminin consists of 6 mg/mL laminin I and Cultrex Collagen of 5 mg/mL rat tail collagen I. Hence, from the three Cultrex hydrogels, Cultrex BME had the highest protein concentration, followed by Cultrex Laminin and Cultrex Collagen. This would not indicate a correlation between higher protein concentration and stiffness and porosity since Cultrex Laminin had the lowest stiffness and Cultrex Collagen the highest. PuraMatrix, a 16-peptide monomer in 0.1–1.0% w/v in water (in our case 0.1%, because this is recommended for neural cells), but does not include any of the typical ECM proteins such as collagen and laminin.^59^ A study by Zhang et al.^60^ suggests that using PuraMatrix with higher w/v increases the fiber and pore size, and it would be interesting to investigate how the cells would react to this, especially with added laminin. Novatach is a sodium alginate grafted with the RGD peptide sequence (GRGDSP) with a guluronic acid content of more than 60%. The material analyses revealed that Novatach did not properly gel. Per the manufacturer’s recommendation, the addition of cell culture media should have been enough to start the gelation process. Adding additional Ca^2+^ ions would have most likely increased gelation and by that also matrix stiffness;^61^ however, as we followed the manufacturer’s recommendations for all hydrogels for consistency and unbiased comparison, we did not perform the addition of ions. It would be an interesting experiment in the future to investigate how various ion concentrations would affect the cells.

## Conclusions

To summarize, our experiments showed that increased laminin concentrations promote elongation of SCs and FBs, but that there is no correlated effect on directed cell migration. Rather, it was found that the combination of laminin and the three-dimensional matrix of hydrogels makes up a unique fibrous and porous matrix structure favorable for oriented, collective migration. Additionally, this combination of pores and fibers in micrometer range seems to enable strain-stiffening, giving the matrix the plasticity to react to deformations. This study revealed the opportunity to make use of the mechanical properties of hydrogels in order to provide optimal structure that favor phenotypes of neurons, SCs, and FBs for PNR. This is invaluable information for the future application of hydrogels in peripheral nerve repair. Only by identifying desirable traits can we achieve a tailored fabrication of biomaterials for successful and, specific utilization, the ultimate goal in tissue engineering.

## Materials and Methods

### Cell Isolation and Culture

Sciatic nerves and DRGs were harvested from adult male Sprague–Dawley rats. The sacrifice of animals was conducted in compliance with the Austrian Animal Testing Law (TVG 2012, §2, 1.c) and Article 3 of the Directive 2010/63/EU of The European Parliament and of the Council on the Protection of Animals Used for Scientific Purposes. SCs and FBs were isolated and cultured as described before.^62−64^ To separate FBs from SCs, we took advantage of the different adhesion properties of SCs and FBs and used a two-step enrichment procedure established by Weiss et al.^56^ After separation, FBs were cultured in MEM∝ supplemented with 10% fetal calf serum (LINARIS), 1% penicillin–streptomycin (P/S, GIBCO), 1% sodium pyruvate solution (GIBCO), and 2.5% 4-(2-hydroxyethyl)-1-piperazineethanesulfonic acid buffer solution (Sigma) on uncoated dishes, while SCs were cultured on 4.8 μg/mL PLL/laminin-coated culture dishes in SC expansion medium according to.^56^ Cells were passaged upon reaching 80–90% confluency and used up to the fifth passage. SC and FB cultures used for experimentation had a purity of over 80% and were cryopreserved in liquid nitrogen.

The harvested DRGs were cultured as described before^26^ in medium consisting of Neurobasal-A medium supplemented with 10 ng/mL recombinant NGF (Invitrogen), 1× B27 supplement (Invitrogen), 2 mM l-glutamine (Invitrogen), and 1% P/S. Medium was changed three times a week. DRG neurons were cultured on 0.01% PDL (Sigma-Aldrich) and 4.8 μg/mL of laminin-coated dishes.

### Preparation of Hydrogels

The synthetic, polypeptide hydrogel PuraMatrix (Corning), the basement membrane extract hydrogel Cultrex 3D Cell Culture Matrix Basement Membrane Extract Reduced Growth Factor (Trevigen), the laminin hydrogel Cultrex 3D Cell Culture Matrix Laminin I, the collagen hydrogel Cultrex 3D Cell Culture Matrix Rat Tail Collagen I, and the alginate peptide-coupled hydrogel Novatach (Novamatrix) were diluted according to the manufacturer’s recommendations. In the case of PuraMatrix, the 1.0% solution was diluted to 0.15% with distilled water. 100 μL of 0.15% PuraMatrix was added into 8-well chamber slides (ibidi) 1 h prior to cell seeding. 100 μL cell culture medium was added and changed three times during this hour in order to establish the recommended pH value. 100% Cultrex 3D Cell Culture Matrix ME, Cultrex 3D Cell Culture Matrix Laminin I, and Cultrex 3D Cell Culture Matrix Rat Tail Collagen I were added to culture dishes 30 min prior to cell seeding. Novatach MVG GRGDSP (delivered as 100 mg of pure lyophilizate) was reconstituted with 5 mL of distilled water resulting in a 2.0% stock solution. 100 μL of this solution was added into the cell culture dish right before cell seeding.

### Seeding Process

For the process of seeding, 100 μL of cell suspension containing either 1.5 × 10^4^ SCs, 1.5 × 10^4^ FBs, or about 50 DRG neurons were added on top of the 100 μL hydrogel or on culture dishes, respectively. As controls (CTRL) served cell-specific coatings, namely, PDL/laminin for the DRG cultures, PLL/laminin for the SCs, and uncoated dishes for the FB cultures. Phase contrast micrographs were obtained daily with a phase-contrast microscope (NIKON Eclipse Ts2R).

### Immunofluorescence Stainings

#### Live/Dead Staining

For the Live/Dead staining, 2 mg/mL of PI solution was added to the cell cultures for 30 min. Subsequently, the cells were washed, fixed, and stained as previously described.^56,64,65^ In short, additionally to PI, SC cultures were stained with SOX10, vimentin (VIME), and DAPI, while FB cultures were stained with THY1, VIME, and DAPI. DRG neuron cultures were stained with TUJ1, VIME, and DAPI. Primary and secondary antibodies are listed in Table S1. Micrographs of the stained cells were taken with a confocal laser scanning microscope (LEICA SP8X). For quantification of PI^+^ dead cells, at least 300 DAPI^+^ nuclei per condition and donor were counted excluding burst cells. The fraction of PI^+^/DAPI^+^/SOX10^+^ was calculated as dead SC, while PI^+^/DAPI^+^/THY1^+^ cells were determined as dead FBs.

#### Cell Proliferation and Culture Purity Assay

In order to determine the mean culture purities for SCs and FBs in percent, cell cultures were stained as previously described.^56,64,65^ Briefly, SC cultures were stained for S100, VIME, and DAPI, while FB cultures were stained for THY1, NGFR, and DAPI. S100^+^/VIME^+^ SCs were differentiated from S100^–^/VIME^+^ FB, while THY^+^/NGFR^–^ FBs were distinguished from THY1^–^/NGFR^+^ SCs. Primary and secondary antibodies are listed in Table S1. Micrographs of the stained cells were taken with a confocal laser-scanning microscope (LEICA SP8X). At least 300 cells per condition and donor were counted.

For the assessment of proliferation rates, 10 μM 5-ethynyl-2′deoxyuridine (EdU, Invitrogen) was added to the SC and FB cultures for 2 h. EdU detection was performed before the immunofluorescence staining steps using Click-iT Plus EdU Alexa Fluor 555 imaging kit (Invitrogen) according to the manufacturer’s protocol. Micrographs of the stained cells were taken with a confocal laser-scanning microscope (LEICA SP8X). To determine the number of proliferating cells in culture, S100^+^/VIME^+^/EdU^+^ SCs and THY^+^/NGFR^–^/EdU^+^ FBs were determined. At least 300 DAPI^+^ nuclei per condition and donor were counted excluding burst cells.

#### Cell Morphology

Using ImageJ, the length (longest line from tip to end of cell) and width (perpendicular line to length) of 20 SCs and FBs per condition and donor were measured manually, and the length/width ratio was calculated.

From DAPI stainings, nucleus size as well as nucleus roundness from SC and FB cultures was quantified. For this, 200 cells of each condition (in total 800 cells per donor) were automatically evaluated using the basic functions of ImageJ (“Analyze particles”). For the DRG neurons, 20 cells per conditions were marked manually and then analyzed using ImageJ. Burst cells as well as cells cut by image frame were excluded.

#### DRG Neuron Characteristics

DRG neuron cultures were stained for neuron marker TUJ1, S100, VIME, and DAPI as previously described.^26^

##### Number of Primary Neurites

For each condition, 20 DRG neuron cell bodies (TUJ1^+^/DAPI^+^ cells) were identified, and the number of primary neurites was counted manually (in total of 80 neurons per donor). Primary neurites are the processes that directly protrude from the cell body. Consequently, the average number of primary neurites per condition was calculated.

##### Number of Branching Points per 500 μm of Neurite

For each condition, 20 DRG neuron cell bodies (TUJ1^+^/DAPI^+^ cells) were identified (in total 80 neurons per donor). The thickest primary neurite was measured, and the number of BPs on that length counted manually. Consequently, the average number of BPs per 500 μm neurite length per condition could be determined.

##### Neurite Thickness

In order to determine the mean neurite thickness, the thickness of the neuron processes was measured at 50 random positions per condition. These random positions had to be on primary neurites from DRGs and within 100 μm from the cell body. Consequently, the mean neurite thickness per condition could be calculated.

In order to evaluate SC/DRG overlap, the overlay of TUJ1 with S100 was analyzed by means of a custom evaluation algorithm (Wolfram Mathematica). The images are weighted so their difference is minimal. From this, the correlation coefficient was calculated as the fraction of the weighted difference between the TUJ1 and S100 over the weighted average.

### Live Cell Imaging

24 h after seeding, live cell imaging of SC and FB cultures in hydrogels and controls was performed with an Olympus IX83 microscope equipped with a stage-top incubator. Using the software cellSens (Olympus Corporation), a picture was taken every 10 min for 17 h. The resulting videos and .tiff stacks were analyzed with ImageJ 1.47 and various plugins. The Manual Tracking plugin was used to track selected cells. 40 cells for each condition were randomly selected (in total 160 cells per donor) and followed throughout the 102 pictures. Afterward, the results were evaluated with ibidi Chemotaxis and Migration Tool, which allowed for the calculation of an average velocity, average total and Euclidean (effective) distance, as well as the directionality index for each cell.

### Laminin Concentration Series

SCs and FBs were seeded on increasing laminin concentration coatings and on PLL and uncoated wells as controls, respectively. For this, 10, 100, and 1000 μg/mL laminin was left on the cell culture well at 37 °C and 5% CO_2_ overnight. 1.0 × 10^4^ SCs and 1.0 × 10^4^ FBs were seeded onto the wells. On the next day, live cell imaging was performed for 17 h, after which the cells were fixated with 4.5% formaldehyde for 15 min on room temperature. Subsequently, SCs were stained for NGFR, phalloidin (Thermo Fisher), and DAPI, while FBs were stained for THY1, phalloidin, and DAPI. Micrographs were taken using a confocal microscope (LEICA SP8X and NIKON Eclipse Ts2R).

### Statistical Analysis

Statistical analysis of logarithmic values between experimental conditions was carried out, and the values were estimated with R-package multcomp 1.4–12^66^ using a two-way ANOVA approach followed by Tukey all-pair comparisons between group means, correcting for the information of the individual cell donors. Results were visualized with GraphPad Prism 8 (GraphPad Software, Inc., La Jolla). All data are presented as mean ± SD and are visible in Table S2.

### Evaluation of Material Properties

#### SEM and AFM

For determination of the morphological characteristics of hydrogels and comparing them to controls, SEM as well as AFM measurements were performed. The hydrogels as well as the laminin concentration series were prepared on glass cover slides. The corresponding coating of each cell type on glass served as controls. Consequently, the cells were seeded and after reaching a high confluency, the cultures were washed once with 1× PBS and fixed with 2.5% glutaraldehyde. Following this, the samples were dehydrated in a sequential series of ethanol (10–30, 50, 70–90, and 3 × 100%) for 20 min each, followed by hexamethyldisilane for 45 min.

For SEM, the samples were coated with 40 nm gold using a sputtercoater (Scancoat Six). A Quanta 250 FEG scanning electron microscope with a secondary electron detector were used to scan randomly chosen positions of the samples.

AFM imaging was performed using a MultiMode Atomic Force Microscope (Ntegra Aura NT-MDT). The images were recorded with tapping mode in ambient conditions using standard tapping mode cantilevers with a force constant of around 40 N/m (Nanosensors, ATEC-NC-10 and NSG03, NT-MDT). Afterward, scans were analyzed using the software Gwyddion (GNU General Public License. Version 2.61). Background correction was performed with a polynomial function, and artefacts were removed using the function “stepline correction” and “remove scars”. Micrographs were exported with adjusted *z*-scales to allow for easier comparison. From these, surface area and roughness were calculated using Gwyddion’s “statistical quantities tool”.

#### Rheological Analyses

A rheometer (Physica MCR 301, Anton Paar, Graz, Austria) was used to obtain the elastic shear modulus (*G*′) and the viscous shear modulus (*G*″) of the hydrogels. A tempered hood was put on top of the measuring system, and a silicone oil-filled evaporation blocker prevented sample drying. Temperature was Peltier controlled and set to 37 °C. 200 μL of each hydrogel was mixed with 200 μL of medium and placed on a profiled plate (*Ø* 2.5 cm, stainless steel). In the case of PuraMatrix, the pH was measured before and after testing using pH-indicator stripes (MColorpHast, Merck). Tests were started as soon as the gel reached a pH of 7.4, representing cell culture conditions. The top plate was lowered, allowing for a 500 μm gap. To allow for stabilization of the hydrogels in the plate–plate geometry, time sweeps were conducted at constant frequency of 6 rad/s and low shear strain of 0.1%. These tests were completed when *G*′ reached a stabilized value, but were limited to a maximum time of 30 min. Subsequently, a frequency sweep test was performed under a constant low oscillating shear deformation of 0.1% to show the linear behavior of the hydrogels. This was followed by a recent large amplitude oscillatory shear stress test protocol^67^ to obtain the nonlinear deformation of the hydrogels (0.01–1000 Pa on a logarithmic shear stress ramp) at constant 6 rad/s to obtain the nonlinear deformation of the hydrogels.

## Data Availability

All data needed to evaluate the conclusions in the paper are present in the paper and/or the Supporting Information.

## Supporting Information Available

SCs were seeded on uncoated wells, wells coated with laminin and PLL, and wells coated with both PLL and laminin; mean ± SD length to width ratio, mean ± SD Velocity_accum_, mean ± SD Distance_accum_, mean ± SD Velocity_euclid_, and mean ± SD Distance_euclid_; SCs and FBs seeded on increasing laminin concentration; scanning electron microscopy images; list of used primary and secondary antibodies; and summary of results (PDF)

## Abbreviations

AFM: atomic force microscopy
BP: branching point
CNS: central nervous system
CTRL: control
DRG: dorsal root ganglion
ECM: extracellular matrix
FB: fibroblast
NGC: nerve guidance conduit
PDL: poly-d-lysine
PLL: poly-l-lysine
PNI: peripheral nerve injury
PNR: peripheral nerve regeneration
PNS: peripheral nervous system
SC: Schwann cell
SD: standard deviation
SEM: scanning electron microscopy
TERM: tissue engineering and regenerative medicine
