# Supplementary material for: Systematic Comparison of Commercial Hydrogels Revealed That a Synergy of Laminin and Strain-Stiffening Promotes Directed Migration of Neural Cells
Source: ACS Appl Mater Interfaces. 2023 Mar 6;15(10):12678–95. doi: 10.1021/acsami.2c20040 (PMC10020957; doi:10.1021/acsami.2c20040)
Supplement: Supplementary file 1 — am2c20040_si_001.pdf [file am2c20040_si_001.pdf]

Supporting information for

A systematic comparison of commercial hydrogels revealed a synergy of laminin and strain-stiffening promotes directed migration of neural cells

Flavia Millesi[1,2,*], Sascha Mero[1,2], Lorenz Semmler[1,2], Anda Rad[1,2], Sarah Stadlmayr[1,2], Anton Borger[1,2], Paul Supper[1,2], Maximilian Haertinger[1,2], Leon Ploszczanski[3], Ursula Windberger[4], Tamara Weiss[1,2], Aida Naghilou[1,2,5,*], Christine Radtke[1,2,6]

[1] Research Laboratory of the Department of Plastic, Reconstructive and Aesthetic Surgery, Medical University of Vienna, Vienna, Austria

[2] Austrian Cluster for Tissue Regeneration, Austria

[3] Institute for Physics and Materials Science, University of Natural Resources and Life Sciences, Vienna, Austria Decentralized

[4] Decentralized Biomedical Facilities, Core Unit Laboratory Animal Breeding and Husbandry, Medical University Vienna, Vienna, Austria

[5] Department of Physical Chemistry, University of Vienna, Vienna, Austria

[6] Department of Plastic, Reconstructive and Aesthetic Surgery, Medical University of Vienna, Vienna, Austria

* corresponding authors; flavia.millesi@meduniwien.ac.at and aida.naghilou@meduniwien.ac.at

Supplementary Figure 1

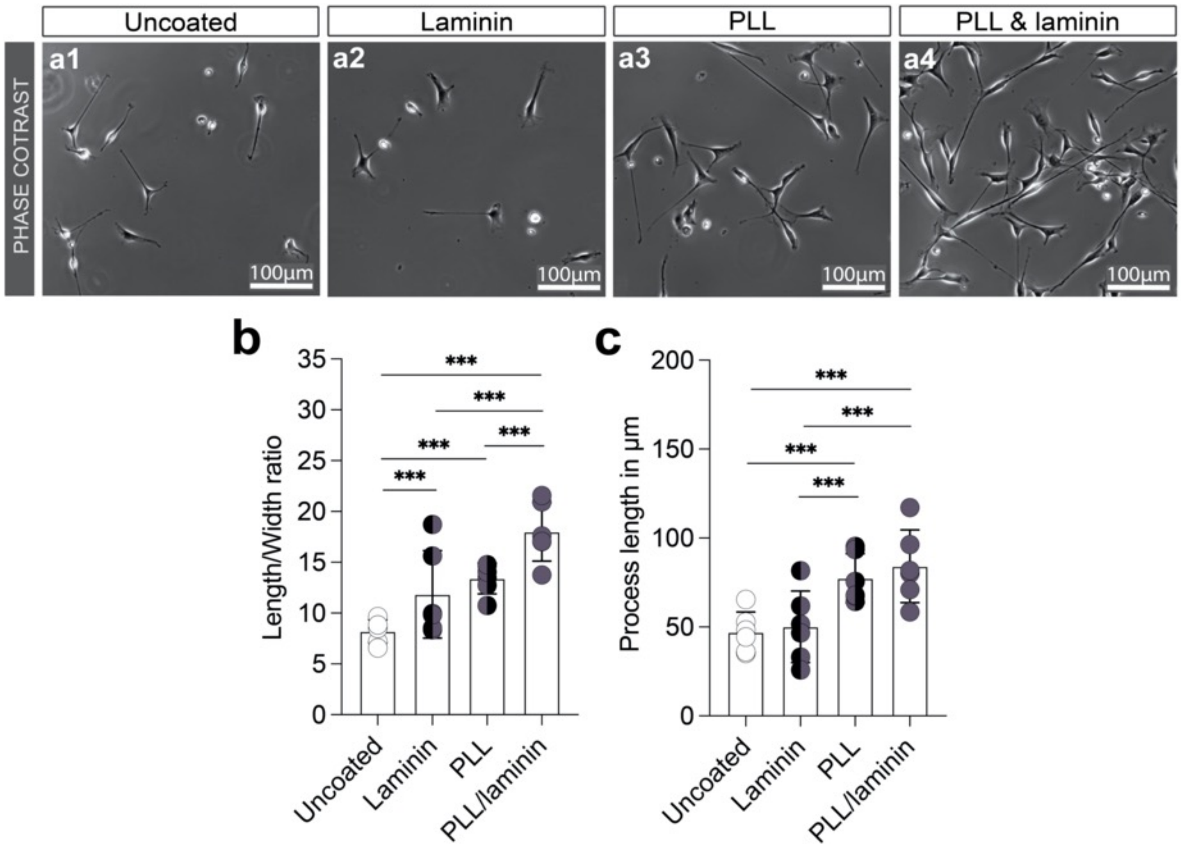

Fig. S1) a SCs were seeded on 1) uncoated wells, wells coated with 2) laminin and 3) PLL respectively as well as on wells coated with 4) both PLL and laminin **b** Diagram depicts the mean ± SD length to width ratio (n=5). **c** Diagram depicts the mean ± SD length of processes in µm (n=5).

Supplementary Figure 2

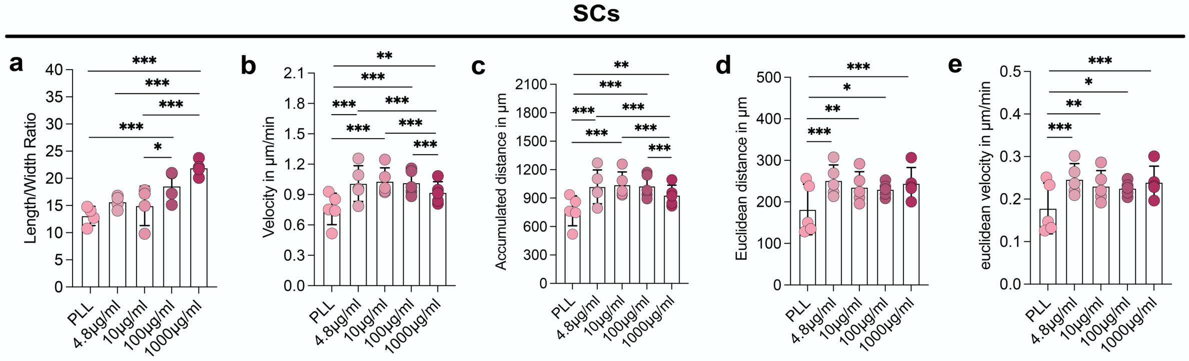

Fig. S2) a Diagram depicts the mean±SD length to width ratio (n=5). **b** Diagram depicts the mean±SD $Velocity_{accum}$ in µm/min (n=5). **c** Diagram depicts the mean±SD $Distance_{accum}$ in µm (n=5). **d** Diagram depicts the mean±SD $Velocity_{euclid}$ in µm/min (n=5). **e** Diagram depicts the mean±SD $Distance_{euclid}$ in µm (n=5).

Supplementary Figure 3

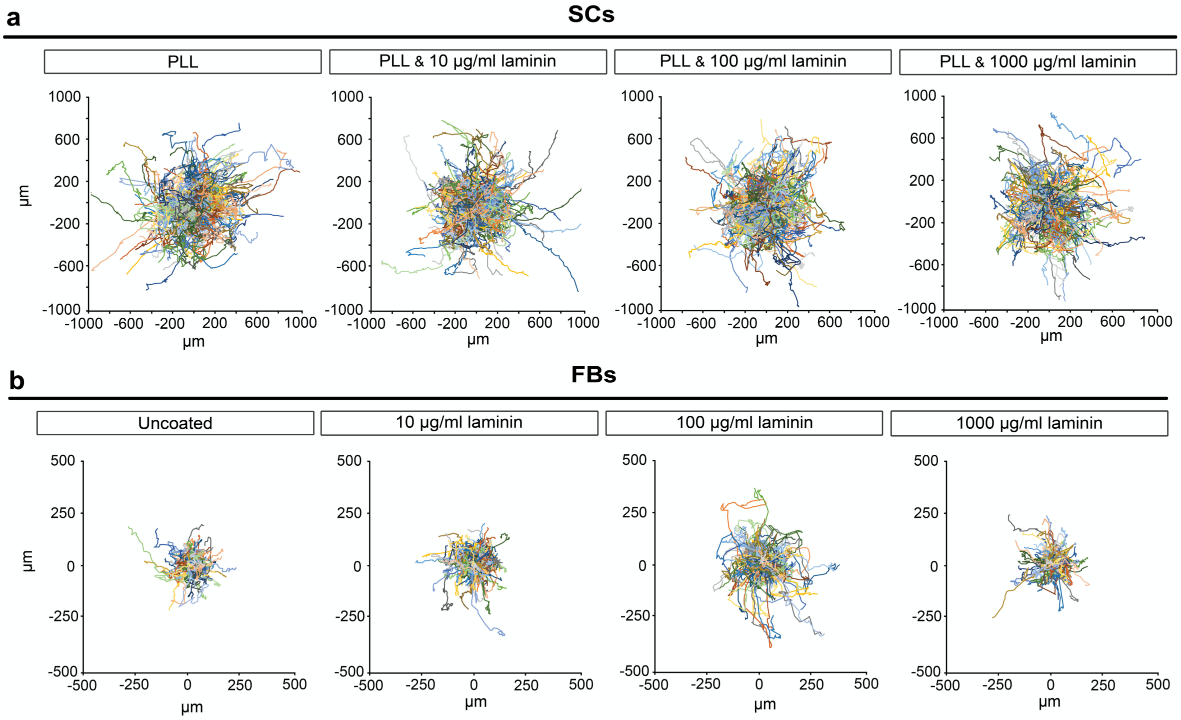

Fig. S3) SCs (a) and FBs (b) seeded on increasing laminin concentration. The colored line in the coordinate system represents each cell starting at 0 (center) for each condition.

Supplementary Figure 4

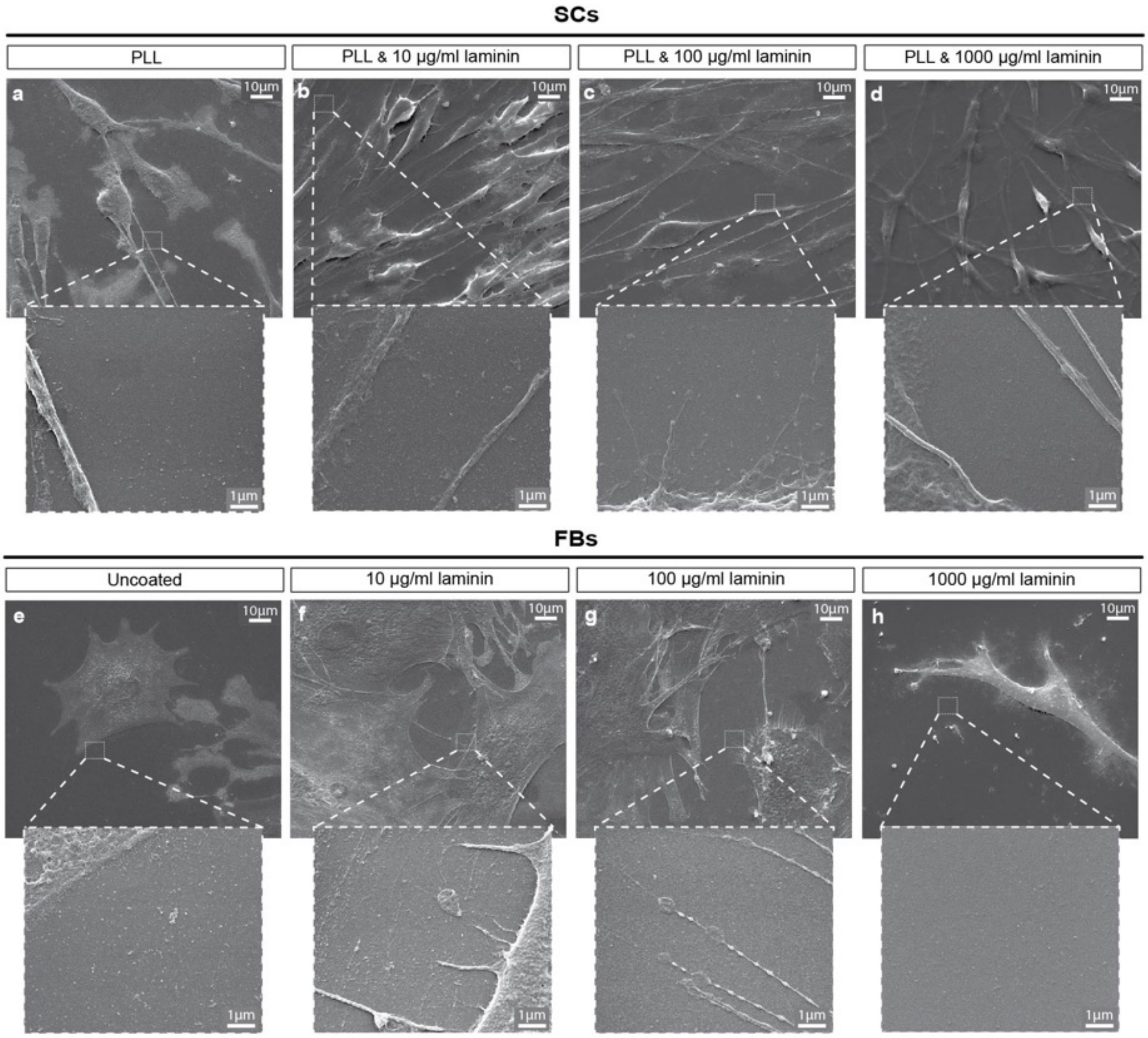

Fig. S4) Scanning electron microscopy of cells on coating with increasing laminin concentrations. Scanning electron micrographs in 2000x and 30000x magnifications of SCs on **a**) PLL, **b**) PLL and 10µg/ml laminin, **c**) PLL and 100µg/ml laminin and **d**) PLL and 1000µg/ml laminin and of FBs on **e**) uncoated wells, and wells coated with **f**) 10µg/ml laminin, **g**) 100µg/ml laminin and **h**) 1000µg/ml laminin.

Supplementary Table 1

Table S1) List of used primary and secondary antibodies.

Primary Antibodies			
Antigen	*species*	*Dilution*	*Company*
S100	rabbit	1:400	DAKO, #Z0311
Vimentin (VIME)	chicken	1:400	ThermoFisher, #PA1-10003
β-3-tubulin (TUJ1)	mouse	1:400	Santa Cruz, sc-51670
THY1	mouse	1:400	Santa Cruz, sc-53116
NGFR	rabbit	1:400	Cell Signalling. #8238S
Secondary Antibodies			
Fluorophore	*Target species*	*Dilution*	*Company*
AF488P	rabbit	1:800	Invitrogen, #A11029
AF594	mouse	1:400	ThermoFisher, #T2767
DL650	chicken	1:400	ThermoFisher, #A11056
AF488	mouse	1:400	ThermoFisher, #A-11029

Supplementary table 2

Table S2) Summary of results in form of means and standard deviations.

Material properties		
Atomic Force Microscopy		
Group	*Surface Roughness [nm]*	*Surface area [µm²]*
FB CTRL	2.95±0.36	104.10±0.94
SC CTRL	1.93±0.34	104.80±1.47
DRG CTRL	3.57±0.49	102.50±1.35
PuraMatrix™	43.75±8.47	118.80±8.289
Cultrex® BME	118.50±20.76	143.10±3.66
Novatach™	8.14±0.47	102.70±1.62
Cultrex® Lam	121.90±6.35	133.30±1.86
Cultrex® Coll	63.37±6.32	117.00±0.76
Rheological Analyses		
Group	*G' [Pa]*	*G'' [Pa]*
PuraMatrix™	14.53±4.13	1.34±0.18
Cultrex® BME	9.47±1.78	0.83±0.07
Novatach™	0.20±0.33	2.54±3.57
Cultrex® Lam	1.11±0.28	0.33±011
Cultrex® Coll	11.55±2.09	2.78±0.24

Nuclei Characteristics		
Schwann Cells		
Group	*Nuclear Area [µm²]*	*Nuclear Roundness (1 = perfectly round)*
CTRL	113.30±20.13	0.63±0.03
PuraMatrix™	118.70±8.97	0.64±0.02
Cultrex®	96.16±17.07	0.56±0.04
Novatach™	133.50±37.90	0.65±0.03
Group	*Nuclear Area [µm²]*	*Nuclear Roundness (1 = perfectly round)*
CTRL	152.00±12.55	0.56±0.03
Cultrex® BME	166.40±15.82	0.53±0.02
Cultrex® Lam	153.30±8.12	0.43±0.02
Cultrex® Coll	125.90±10.85	0.63±0.1
Fibroblasts		
Group	*Nuclear Area [µm²]*	*Nuclear Roundness (1 = perfectly round)*
CTRL	215.50±35.24	0.69±0.03
PuraMatrix™	205.10±32.46	0.69±0.02
Cultrex®	136.10±33.43	0.64±0.02
Novatach™	206.00±30.69	0.70±0.02
Group	*Nuclear Area [µm²]*	*Nuclear Roundness (1 = perfectly round)*
CTRL	213.00±14.23	0.72±0.03
Cultrex® BME	58.35±0.99	0.63±0.05
Cultrex® Lam	109.40±38.34	0.67±0.20
Cultrex® Coll	136.50±27.25	0.65±0.44

DRG Neurons		
Group	*Nuclear Area [μm^2]*	*Nuclear Roundness (1 = perfectly round)*
CTRL	166.40±31.35	0.83±0.05
PuraMatrix™	143.70±20.08	0.82±0.03
Cultrex®	114.30±10.24	0.85±0.04
Novatach™	134.40±14.20	0.86±0.01
Group	*Nuclear Area [μm^2]*	*Nuclear Roundness (1 = perfectly round)*
CTRL	103.30±21.55	0.77±0.05
Cultrex® BME	74.04±5.54	0.78±0.04
Cultrex® Lam	85.26±3.34	0.78±0.05
Cultrex® Coll	96.03±7.72	0.86±0.02

**Cell Condition**				
Schwann Cells				
Group	*% SCs in culture*	*% Proliferating cells*	*% alive cells*	*Length/width ratio*
CTRL	99.64±0.44	35.28±10.41	99.36±0.70	
PuraMatrix™	99.19±1.19	21.36±9.661	98.92±1.05	
Cultrex®	99.78±0.31	16.84±11.57	98.15±0.96	
Novatach™	99.61±0.22	24.98±8.733	99.18±0.69	
Group	*% Proliferating cells*	*Length/width ratio*		
CTRL	24.75±12.49	14.44±4.87		
Cultrex® BME	17.50±12.61	19.55±7.19		
Cultrex® Lam	20.07±5.57	23.73±10.00		
Cultrex® Coll	0.79±1.57	7.09±2.57		
Group	*Length/width ratio*			
PLL	13.08 ±1.76			
10 μg/ml Lam	14.93±3.63			
100 μg/ml Lam	18.56±2.89			
1000 μg/ml Lam	21.90±1.49			
Fibroblasts				
Group	*% FBs in culture*	*% Proliferating cells*	*% alive cells*	*Length/width ratio*
CTRL	91.81±7.26	7.68±3.62	99.16±0.50	
PuraMatrix™	91.19±8.87	5.81±4.68	99.23±0.46	
Cultrex®	89.12±6.32	2.24±2.10	98.92±0.58	
Novatach™	91.57±12.55	3.50±3.88	98.51±0.93	
Group	*% Proliferating cells*	*Length/width ratio*		
CTRL	4.64±4.00	2.57±0.32		
Cultrex® BME	1.82±0.63	6.53±1.13		
Cultrex® Lam	4.05±1.21	7.21±1.91		
Cultrex® Coll	0.00±0.00	9.48±0.51		
Group	*Length/width ratio*			
Uncoated	3.27±0.52			
10 μg/ml Lam	5.56±1.60			
100 μg/ml Lam	5.75±0.74			

1000 µg/ml Lam	15.88±4.300			

DRG Neurons				
Group	*% Overlap TUJ1/S100*	*# Primary neurites*	*# BPs/500µm*	*Neurite Thickness [µm]*
CTRL	38.85±12.97	2.39±0.54	1.19±0.46	1.35±0.29
PuraMatrix™	35.05±7.490	2.53±0.36	2.12±1.54	1.50±0.39
Cultrex®	63.85±4.226	1.20±0.18	1.06±0.59	1.21±0.15
Novatach™	30.92±1.543	2.75±0.15	1.79±1.14	1.15±0.13
Group	*% Overlap TUJ1/S100*	*# Primary neurites*	*# BPs/500µm*	*Neurite Thickness [µm]*
CTRL	51.30±5.56	1.96±0.34	1.79±0.70	0.95±0.30
Cultrex® BME	84.32±6.18	1.21±0.11	1.38±0.38	1.03±0.27
Cultrex® Lam	79.57±1.93	1.20±0.10	2.07±1.33	0.74±0.15
Cultrex® Coll	81.67±1.86	1.90±0.17	1.25±0.10	0.68±0.11

Migration				
Schwann Cells				
Group	*Accumulated Velocity [µm/min]*	*Accumulated Distance [µm]*	*Euclidean Distance [µm]*	*Euclidean Velocity [µm/min]*
CTRL	0.83±0.11	842.70±115.30	163.30±55.50	0.16±0.05
PuraMatrix™	0.29±0.028	298.60±30.03	61.88±19.17	0.06±0.01
Cultrex®	0.62±0.13	630.70±130.60	249.80±39.48	0.24±0.03
Novatach™	0.71±0.11	726.20±113.10	184.50±27.02	0.18±0.02
Group	*Accumulated Velocity [µm/min]*	*Accumulated Distance [µm]*	*Euclidean Distance [µm]*	*Euclidean Velocity [µm/min]*
CTRL	0.98±0.27	989.10±266.60	182.4±64.44	0.18±0.06
Cultrex® BME	0.81±0.26	815.30±261.60	249.8±32.72	0.24±0.03
Cultrex® Lam	0.52±0.15	499.50±186.70	238.6±75.77	0.23± 0.07
Cultrex® Coll	0.67±0.24	647.60±278.30	200.7±58.15	0.20±0.06
Group	*Accumulated Velocity [µm/min]*	*Accumulated Distance [µm]*	*Euclidean Distance [µm]*	*Euclidean Velocity [µm/min]*
PLL	0.76±0.16	765.50±157.60	181.50±60.70	0.18.±0.60
10 µg/ml Lam	1.03±0.14	1039.00±136.50	234.60±37.94	0.23±0.04
100 µg/ml Lam	1.02±0.12	1025.00±124.70	229.70±16.91	0.23±0.02
1000 µg/ml Lam	0.92±0.11	927.50±109.00	244.10±38.79	0.24±0.04
Fibroblasts				
Group	*Accumulated Velocity [µm/min]*	*Accumulated Distance [µm]*	*Euclidean Distance [µm]*	*Euclidean Velocity [µm/min]*
CTRL	0.20±0.02	206.90±30.08	52.90±5.24	0.05±0.01
PuraMatrix™	0.30±0.02	304.70±25.58	75.22±10.95	0.07±0.01
Cultrex®	0.19±0.02	201.60±28.86	123.30±42.75	0.12±0.04
Novatach™	0.20±0.03	210.70±34.24	54.57±9.59	0.05±0.01
Group	*Accumulated Velocity [µm/min]*	*Accumulated Distance [µm]*	*Euclidean Distance [µm]*	*Euclidean Velocity [µm/min]*
CTRL	0.21±0.06	217.40±60.27	46.70±4.80	0.05±0.004
Cultrex® BME	0.14±0.04	137.90±38.47	65.67±19.69	0.06±0.02
Cultrex® Lam	0.16±0.01	164.60±13.01	67.44±11.20	0.07±0.01

Cultrex® Coll	0.16±0.06	159.3±60.81	36.43±11.96	0.04±0.01
Group	*Accumulated Velocity [µm/min]*	*Accumulated Distance [µm]*	*Euclidean Distance [µm]*	*Euclidean Velocity [µm/min]*
Uncoated	0.20±0.02	206.20±19.45	47.77±7.87	0.05±0.01
10 µg/ml Lam	0.25±0.03	250.30±27.43	60.36±5.71	0.06±0.01
100 µg/ml Lam	0.32±0.03	319.00±28.95	84.90±10.18	0.08±0.01
1000 µg/ml Lam	0.20±0.02	203.30±23.56	48.77±9.50	0.05±0.01
